# Exploring the neuroprotective effects and underlying mechanisms of medical cannabinoids in ischemic stroke: a systematic meta-analysis with bibliometric mapping of cerebral ischemia research

**DOI:** 10.3389/fnins.2025.1731738

**Published:** 2026-01-02

**Authors:** Xiaoqun Li, Lulu Wen, Yun Du, Xinyue Fan, Ningyu Xue, Miao Qu

**Affiliations:** 1 Third Clinical Medical College of Beijing University of Chinese Medicine, Beijing, China; 2 Department of Neurology, Xuanwu Hospital of Capital Medical University, Beijing, China; 3 Department of Chinese Medicine, Xuanwu Hospital of Capital Medical University, Beijing, China

**Keywords:** bibliometric analysis, ischemic stroke, MCAO, medical cannabinoids, meta-analysis, neuroprotective mechanisms

## Abstract

**Background:**

Ischemic stroke is an acute neurological disorder with limited treatment options. Medical cannabinoids (MCs), primary bioactive compounds extracted from cannabis plants, have shown therapeutic prospects for ischemic stroke. This study integrates bibliometrics and meta-analysis to comprehensively summarize the research landscape of MCs in cerebral ischemia and thoroughly investigate their role and potential mechanisms in ischemic stroke.

**Methods:**

Bibliometric analysis was performed based on literature retrieved from Web of Science Core Collection (WoSCC), PubMed, and Scopus. For meta-analysis, a comprehensive search was conducted across four databases (WoSCC, PubMed, Embase, and Cochrane Library) and grey literature repositories. Studies were screened according to predefined criteria. Pooled standardized mean differences with 95% confidence interval were calculated, followed by subgroup analysis.

**Results:**

A total of 241 publications were identified for bibliometric analysis. From 2000 to June 2025, the annual publication output on MCs in cerebral ischemia displayed a fluctuating yet overall upward trend. Keyword co-occurrence analysis revealed three major research topics: neuroprotective mechanisms of MCs, pathological models of cerebral ischemia, and bioactive components of MCs. Meta-analysis of 26 studies demonstrated that MCs provided significant neuroprotection in animal models of ischemic stroke, including cerebral infarct volume, neurological function score (NFS), cerebral blood flow (CBF), blood–brain barrier (BBB) permeability, brain water content, apoptosis (TUNEL-positive cells), oxidative stress markers, inflammation (TNF-*α*, IL-1β), and excitotoxicity (Glu/NAA, Lac/NAA ratio). Subgroup analysis revealed that intraperitoneal administration and a full-course of cannabidiol (CBD) treatment were associated with reduced heterogeneity and enhanced therapeutic benefit. Isoflurane was identified as a potentially suitable anesthetic.

**Conclusion:**

MCs exert multi-target neuroprotection in ischemic stroke by improving CBF, reducing brain edema and BBB permeability, and inhibiting oxidative stress, neuroinflammation, apoptosis, and excitotoxicity. Future research should focus on high-quality clinical trials to validate these findings and translate MCs into clinical practice.

**Systematic review registration:**

https://osf.io/6je7n.

## 1 Introduction

Cerebral stroke is one of the leading causes of high mortality and disability worldwide, with a lifetime risk as high as 25% (Feigin et al., 2018). As of 2021, there were 93.8 million cerebral stroke patients globally, resulting in annual disease costs exceeding $890 billion and imposing a significant economic burden on society and families (GBD 2021 Stroke Risk Factor Collaborators, 2024). Among the subtypes of stroke, ischemic stroke accounts for approximately 65 to 87% (Feigin et al., 2025). Its primary pathological characteristic is cerebrovascular occlusion (due to atherosclerosis, thrombosis, or embolus detachment), which leads to cerebral ischemia and hypoxia. This subsequently triggers a series of cascading reactions, ultimately resulting in neuronal injury and neurological deficits (Deng et al., 2025). Rapid restoration of blood flow to the affected brain regions is crucial for the treatment of ischemic stroke. Current treatments mainly include intravenous thrombolysis and endovascular interventions, with therapeutic windows of 4.5 h and 6 h after symptom onset, respectively. Tissue-type plasminogen activator (tPA) is the only thrombolytic agent approved by the Food and Drug Administration (FDA) for clinical use. The use of this drug is strictly time-limited, and administration outside the therapeutic window increases the risk of intracranial hemorrhage (Barthels and Das, 2020). Additionally, interventional therapies such as mechanical thrombectomy have well-defined indications. As potential adjunctive therapies for ischemic stroke, neuroprotective agents (e.g., edaravone, butylphthalide, vinpocetine) face significant challenges, including a narrow therapeutic time window and poor blood–brain barrier (BBB) penetration (Wang et al., 2023; Wang et al., 2024; Chen et al., 2025). Consequently, their clinical efficacy remains to be fully proven and requires confirmation in large-scale, multicenter, high-quality clinical trials. Therefore, the current treatments for ischemic stroke are relatively limited, and it is necessary to seek new promising therapeutic strategies.

With the continuous revelation of the medical value of cannabinoids, an increasing number of countries globally have approved cannabinoids for clinical treatment (Hidding et al., 2024; Hoch et al., 2025). Cannabinoids are primarily categorized into phytocannabinoids, endocannabinoids, and synthetic cannabinoids. To date, more than 120 phytocannabinoids have been isolated and identified from cannabis plants, among which cannabidiol (CBD) and D-9-tetrahydrocannabinol (THC) are the two most intensively studied components (Stone et al., 2020). Medical cannabinoids (MCs) refer to natural or synthetic cannabinoid compounds that can improve disease states or alleviate symptoms (Whiting et al., 2015). In recent years, the potential value of MCs in the treatment of ischemic cerebrovascular diseases has drawn increasing attention. CBD acts as a negative allosteric modulator of cannabinoid receptors (CBR) and exerts brain-protective effects through multi-target regulatory properties (Raïch et al., 2024). A meta-analysis further revealed that CBD can increase cerebral blood flow (CBF) and decrease arterial blood pressure after stroke (Sultan et al., 2017). By partially activating CB1R and CB2R, THC produces anti-inflammatory effects, reduces neuronal damage, and promotes hippocampal neurogenesis (Suliman et al., 2018; Amin and Ali, 2019). Dronabinol and nabilone, THC-based synthetic medications approved by the FDA, may have limited clinical applications due to their psychoactive effects. Additionally, some studies have suggested that the use of MCs may reduce the risk of ischemic stroke (Scharf, 2017; San Luis et al., 2020). Preclinical studies have revealed that MCs can exert anti-inflammatory, antioxidant, anti-excitotoxic, and anti-apoptotic effects by modulating receptors such as CBR, serotonin (5-HT), transient receptor potential vanilloid channels, and other G protein-coupled receptors, thereby combating ischemic brain injury (Vicente-Acosta et al., 2022; Castillo-Arellano et al., 2023). A comprehensive investigation of the role of MCs in cerebral infarction is of great significance for the treatment and prognosis of stroke.

Bibliometrics enables knowledge visualization through quantitative analysis, thereby revealing hotspots and trends within a specific field. This study conducted a scientometric analysis of the literature on MCs and cerebral ischemia, and utilized meta-analysis to evaluate their specific benefits in animal models of ischemic stroke. This integrated approach aims to systematically elucidate the evolution of the field and explore the therapeutic efficacy and neuroprotective mechanisms of MCs, thereby offering a scientific foundation and reference for future research.

## 2 Methods

### 2.1 Research methods of bibliometrics

A comprehensive search of the Web of Science Core Collection (WoSCC), PubMed, and Scopus databases was conducted to retrieve relevant literature published from 1 January 2000 to 30 June 2025. Our bibliometric search was designed to capture the broad field of cerebral ischemia research. To ensure comprehensiveness, it encompassed studies on both focal and global ischemia, as well as other relevant experimental models. The search strategy was as follows: (medical cannabinoid* OR medical cannabis OR medical marijuana OR cannabidiol OR dronabinol OR nabiximols) AND (ischemic stroke* OR cerebral ischemia OR cerebral stroke* OR cerebrovascular accident* OR cerebral infarct* OR cerebral embolism). Further details are available in Supplementary Table 1. Only articles and reviews published in English were included in this study. Literature that was not relevant to the research topic was excluded. The eligible data were subjected to visual analysis using VOSviewer (1.6.20) (van Eck and Waltman, 2010) and CiteSpace (6.4. R 1) (Chen, 2006). The content of the analysis included annual publication volume, countries, institutions, authors and keywords. The significant cooperative relationships and research hotspots in this field were identified by constructing cooperation networks among countries, institutions, and authors, as well as co-occurrence maps of keywords.

### 2.2 Research methods of meta-analysis

This systematic review and meta-analysis was reported in accordance with the Preferred Reporting Items for Systematic Reviews and Meta-Analyses (PRISMA) statement (Moher et al., 2009). The protocol of this study has been registered on the Open Science Framework platform (DOI: 10.17605/OSF.IO/6JE7N).

#### 2.2.1 Data sources and search strategy

We further conducted a meta-analysis to evaluate the efficacy of MCs in focal ischemic stroke using middle cerebral artery occlusion (MCAO) models. Four databases (WoSCC, PubMed, Embase, and Cochrane Library) were searched from 1 January 2000 to 30 June 2025, with the last search on the latter date. The search strategy combined medical subject headings with free-text terms. Detailed search strategy is provided in Supplementary Table 1. In addition, grey literature databases (Open Grey, bioRxiv, and medRxiv) were searched to identify potentially eligible preprints.

#### 2.2.2 Inclusion and exclusion criteria

The inclusion criteria for this study were as follows: (1) Study type: Published animal experiments; (2) Study subjects: Focal ischemic stroke models induced by MCAO; (3) Intervention type: The experimental group received MCs or their derivatives, while the control group was administered conventional drugs, vehicle treatment, or a blank control; (4) Outcome measures: cerebral infarct volume, neurological function score (NFS), CBF, BBB permeability and brain water content, oxidative stress markers, inflammatory indicators [tumor necrosis factor-*α* (TNF-α), interleukin-1β (IL-1β)], apoptosis indicators [number of terminal deoxynucleotidyl transferase (TdT)-mediated dUTP-biotin nick end labeling (TUNEL)-positive cells], and excitotoxicity index [glutamate/N-acetylaspartate (Glu/NAA) ratio, lactate/N-acetylaspartate (Lac/NAA) ratio]. Exclusion criteria included the following: (1) Reviews, comments, duplicate publications, conference abstracts, case reports, and editorials; (2) Studies that employed other interventions in combination; (3) Studies lacking a control group; (4) Non-English published studies; (5) Studies that did not provide a clear treatment protocol; (6) Studies that failed to report sample size in detail.

#### 2.2.3 Study selection and data extraction

Two evaluators independently conducted literature screening and data extraction processes. After removing duplicates, the preliminary screening was performed by reviewing the titles and abstracts of the articles. Then, full-text reading was carried out to further screen the articles according to the inclusion and exclusion criteria. The screening results from both evaluators were cross-checked for consistency. Finally, all eligible articles were identified, and relevant data were extracted, including title, publication year, author names, study subjects, interventions, and outcome measures. When multiple dosing times or doses were present in the literature, data corresponding to the best therapeutic effect were extracted. To comprehensively assess the intervention efficacy, we contacted corresponding authors via email for studies with data presented only in graphical form to obtain complete information necessary for our analysis. If no response was received, data were extracted with WebPlotDigitizer program. We used the following formula for data transformation: $\mathrm{SD}=\mathrm{SEM}\times \sqrt{n}$ .

#### 2.2.4 Risk of bias assessment

Two evaluators independently assessed the quality of the included studies and drew the risk of bias graph. The assessment of literature quality was conducted using the Systematic Review Center for Laboratory Animal Experimentation (SYRCLE) risk of bias tool, which comprises 10 items (Hooijmans et al., 2014). In case of disagreement during the above process, a third evaluator was invited to resolve the conflicts and achieve consensus.

#### 2.2.5 Data analysis

The effect size of the outcome measures was described as the standardized mean difference (SMD) with 95% confidence interval (CI). A test for heterogeneity among studies was performed to select the corresponding model. If *I^2^* < 50%, the fixed-effects model was used. Otherwise, the random-effects model was applied, accompanied by subgroup analyses to explore potential sources of heterogeneity. The threshold for statistical significance was set at *p* < 0.05. When more than 10 studies were included for a particular outcome measure, funnel plots were constructed and the Egger test was performed to assess publication bias. All data analyses were performed with Review Manager 5.3 (RevMan, Cochrane Collaboration) and Stata 17.0 software.

## 3 Results

### 3.1 Results of bibliometrics

#### 3.1.1 The trend of annual publications

**Figure 1:**
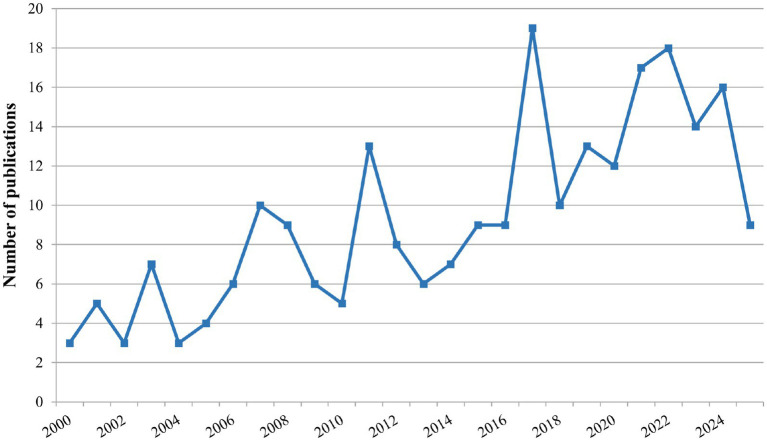
Annual publications trends on research of MCs in cerebral ischemia.

In this study, 241 publications related to the application of MCs in cerebral ischemia were identified, including 144 articles and 97 reviews. The annual publication output is presented in Figure 1. The findings revealed three distinct phases of research activity. From 2000 to 2010, it was the initial stage of research, with low and fluctuating publication output. From 2011 to 2017, it entered a period of rapid growth. Since 2018, it has been a period of stable development, with publication numbers remaining high. In 2025, there was a slight decrease to 9 publications, but we only searched for early publications for that year, and subsequent publication numbers may still increase. In general, from 2000 to June 2025, the number of publications shows an overall upward trajectory with fluctuations, reflecting a sustained growth in researchers’ attention and research interest in this field.

#### 3.1.2 Visualization of scientific collaboration network

**Figure 2:**
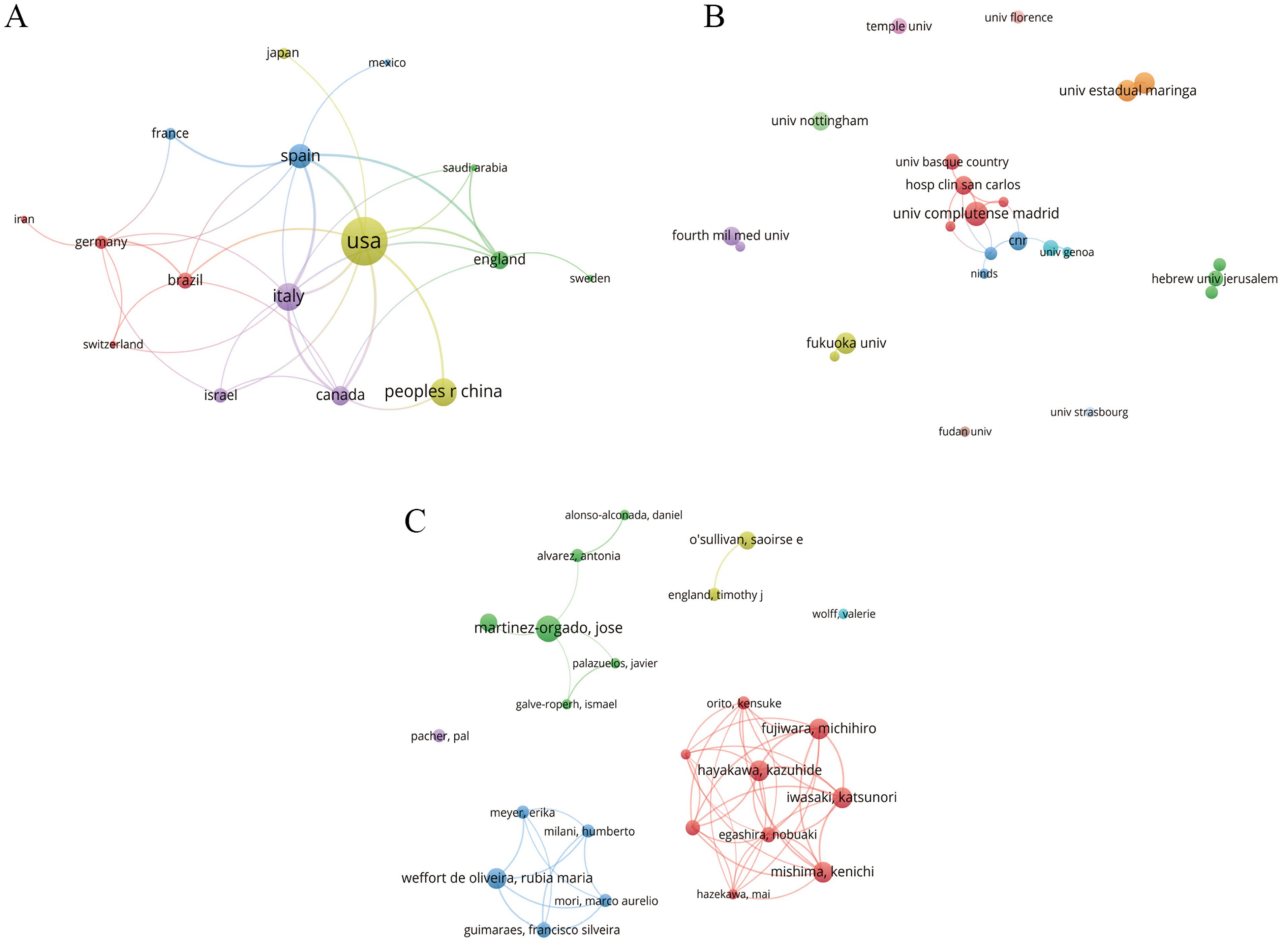
**(A)** Collaboration network among countries/regions. **(B)** Collaboration network among institutions. **(C)** Collaboration network among authors.

The international cooperation network showed that 43 countries or regions were actively engaged in this research field (Figure 2A). The United States ranked first with 74 publications, followed by Italy with 32. The top 10 countries/regions in terms of number of publications are presented in Table 1, accounting for 77.6% of all publications. Notably, the United States led both productivity and the number of collaborators, highlighting its core contributions and preeminent position within this research topic.

**Table 1:** The top 10 countries/regions, institutions, and authors for publications.

Indicator	Rank	Name	Total link strength	Count	Rank	Name	Total link strength	Count
Country/Region	1	The United States	33	74	6	England	13	16
	2	Italy	21	32	7	Brazil	7	14
	3	China	7	31	8	Israel	5	12
	4	Spain	21	26	9	Germany	8	10
	5	Canada	17	18	10	France	4	9
Institution	1	Complutense University of Madrid	35	9	6	Fourth Military Medical University	18	7
	2	Fukuoka University	14	8	7	Hospital Clinico San Carlos	24	7
	3	State University of Maringa	8	8	8	University of Nottingham	4	7
	4	University of São Paulo	6	8	9	Sapienza University of Rome	20	6
	5	Consiglio Nazionale delle Ricerche	16	7	10	University of the Basque Country	16	6
Author	1	Jose Martinez-Orgado	82	10	6	Rubia Maria Weffort de Oliveira	46	8
	2	Michihiro Fujiwara	84	8	7	Vincenzo Di Marzo	50	7
	3	Kazuhide Hayakawa	84	8	8	Saoirse E O’Sullivan	17	7
	4	Katsunori Iwasaki	84	8	9	Nobuaki Egashira	60	6
	5	Kenichi Mishima	84	8	10	Masayuki Fujioka	68	6

A total of 394 institutions worldwide conducted research in related fields (Figure 2B). Table 1 lists the top 10 institutions in terms of contribution. Complutense University of Madrid topped the list in terms of the number of publications and total link strength, demonstrating the greatest influence on research concerning MCs and cerebral ischemia. It is noteworthy that there may not be a direct correlation between research output and collaboration among institutions; those exhibiting lower output may be more active in cooperation. For instance, Hospital Clinico San Carlos and Sapienza University of Rome displayed high total link strengths despite their relatively modest publication counts. In addition, the depth and breadth of cooperation between institutions required further enhancement.

A co-authorship network was constructed with authors as nodes, comprising 1,127 core authors (Figure 2C). Table 1 shows the top 10 most productive authors. The author with the greatest contribution was Jose Martinez-Orgado, who published 10 articles. The academic team centered around Jose Martinez-Orgado occupied a central position in the network, reflecting extensive collaborations and considerable influence. Many other research groups also existed within the author network. The fewer links and greater distance between these teams suggested that current cerebral ischemia research focused more on collaboration within teams rather than across different teams.

#### 3.1.3 Visualization of keyword network

**Figure 3:**
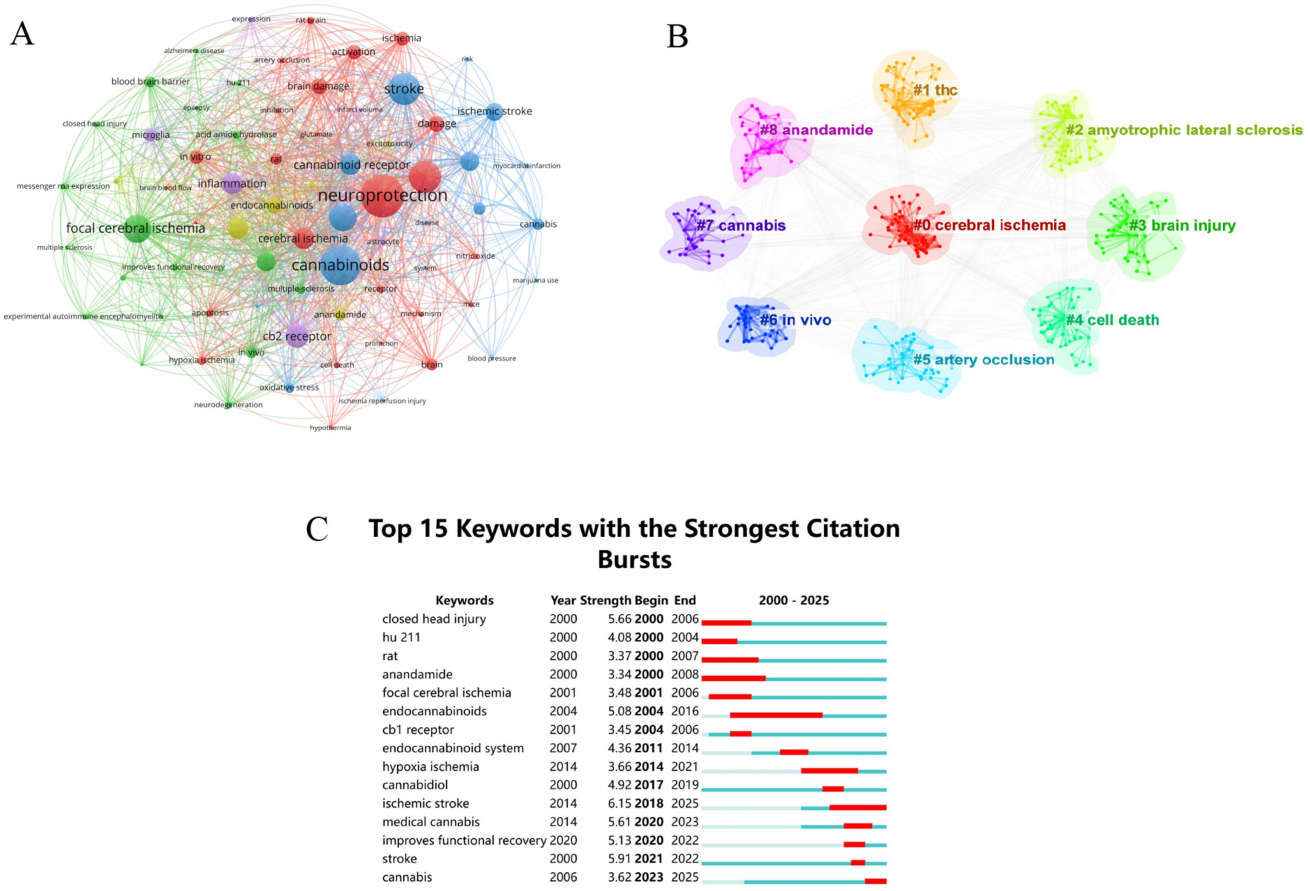
**(A)** The keyword co-occurrence network. **(B)** The keyword cluster network. **(C)** Top 15 keywords with the strongest citation bursts. THC, D-9-tetrahydrocannabinol; cb1 receptor, cannabinoid receptor 1.

Analysis of the keyword co-occurrence network can clearly reveal the current status and hotspots in the research field. Figure 3A shows a keyword network consisting of 1,245 nodes. We compiled the top 20 keywords, as shown in Table 2. The search terms “cannabinoids,” “stroke,” and “cerebral ischemia” ranked high in frequency, reflecting that this field has attracted extensive attention and research interest of investigators. Using the log-likelihood ratio for keyword clustering and visualization analysis, we obtained 9 cluster combinations. In our clustering model (Q = 0.4168, S = 0.7763), both the cluster structure and results were robust. The 9 clusters were labeled as follows: #0 cerebral ischemia, #1 THC, #2 amyotrophic lateral sclerosis (ALS), #3 brain injury, #4 cell death, #5 artery occlusion, #6 *in vivo*, #7 cannabis, and #8 anandamide, as shown in Figure 3B. It is worth noting that the ALS cluster was primarily derived from the review articles included in the analysis. However, there is currently insufficient original research to directly confirm the association between the therapeutic effects of MCs on ALS and the mechanisms of cerebral ischemia. Therefore, this study focused on the core topic clusters related to cerebral ischemia.

**Table 2:** The top 20 keywords.

Rank	Keywords	Total link strength	Occurrences	Rank	Keywords	Total link strength	Occurrences
1	Neuroprotection	1,176	86	11	Inflammation	568	42
2	Cannabinoids	1,086	80	12	Delta 9 tetrahydrocannabinol	513	39
3	Cannabidiol	853	64	13	Animal model	538	38
4	Stroke	841	64	14	Ischemic stroke	431	36
5	Focal cerebral ischemia	786	56	15	Damage	404	32
6	Endocannabinoid system	753	55	16	Brain damage	422	31
7	Cannabinoid receptor 2	665	47	17	Endocannabinoids	430	30
8	Cannabinoid receptor	627	43	18	Activation	379	29
9	Cannabinoid receptor 1	583	42	19	Ischemia	386	28
10	Cerebral ischemia	578	42	20	In vitro	378	27

Combined with the results of keyword co-occurrence and clustering, the research hotspots in this field were summarized into three distinct areas. The keywords “neuroprotection,” “inflammation,” “endocannabinoid system,” “cannabinoid receptor,” and “cell death” revealed the molecular mechanisms through which cannabinoids exert neuroprotective effects and mitigate brain damage after cerebral ischemia. The results related to “animal model,” “brain damage,” “*in vivo*,” and “artery occlusion” showed that the current hot research topics were primarily focused on pathological models and experimental studies. This study encompassed both in vivo animal models and *in vitro* cellular models. The in vivo models included focal cerebral ischemia models [transient middle cerebral artery occlusion (tMCAO), permanent middle cerebral artery occlusion (pMCAO), and photothrombosis model], as well as models for studying global cerebral ischemic injury (two-vessel occlusion, four-vessel occlusion, and hypoxic–ischemic brain damage model). The in vitro models comprised the oxygen–glucose deprivation (OGD) model and the oxygen–glucose deprivation/reoxygenation (OGD/R) model. In treatment, the most widely used cannabinoids were “cannabidiol” and “delta 9 tetrahydrocannabinol.”

Keyword burst analysis can reflect the prevailing topics in a research field during specific periods thereby offering a scientific basis for predicting frontier development trends. The top 15 keywords with the highest burst rates are shown in Figure 3C. In the initial stages the protective effects of the cannabinoid HU-211 in closed head injury and focal cerebral ischemia were primarily investigated with rats being the main experimental subjects. Concurrently attention began to shift towards the role of endocannabinoids (anandamide) in the nervous system. From 2004 to 2014 the overall function and mechanism of the endocannabinoid system became a hot topic. Subsequently research increasingly focused on the manifestation of CBD in ischemic encephalopathy. Over the past 5 years studies on medical cannabis drugs for post-stroke neural function recovery have gradually emerged though still remain at the preclinical stage. This suggests that future trends may prioritize translating preclinical studies of MCs into clinical applications for ischemic stroke.

### 3.2 Results of meta-analysis

#### 3.2.1 Study selection and basic characteristics

**Figure 4:**
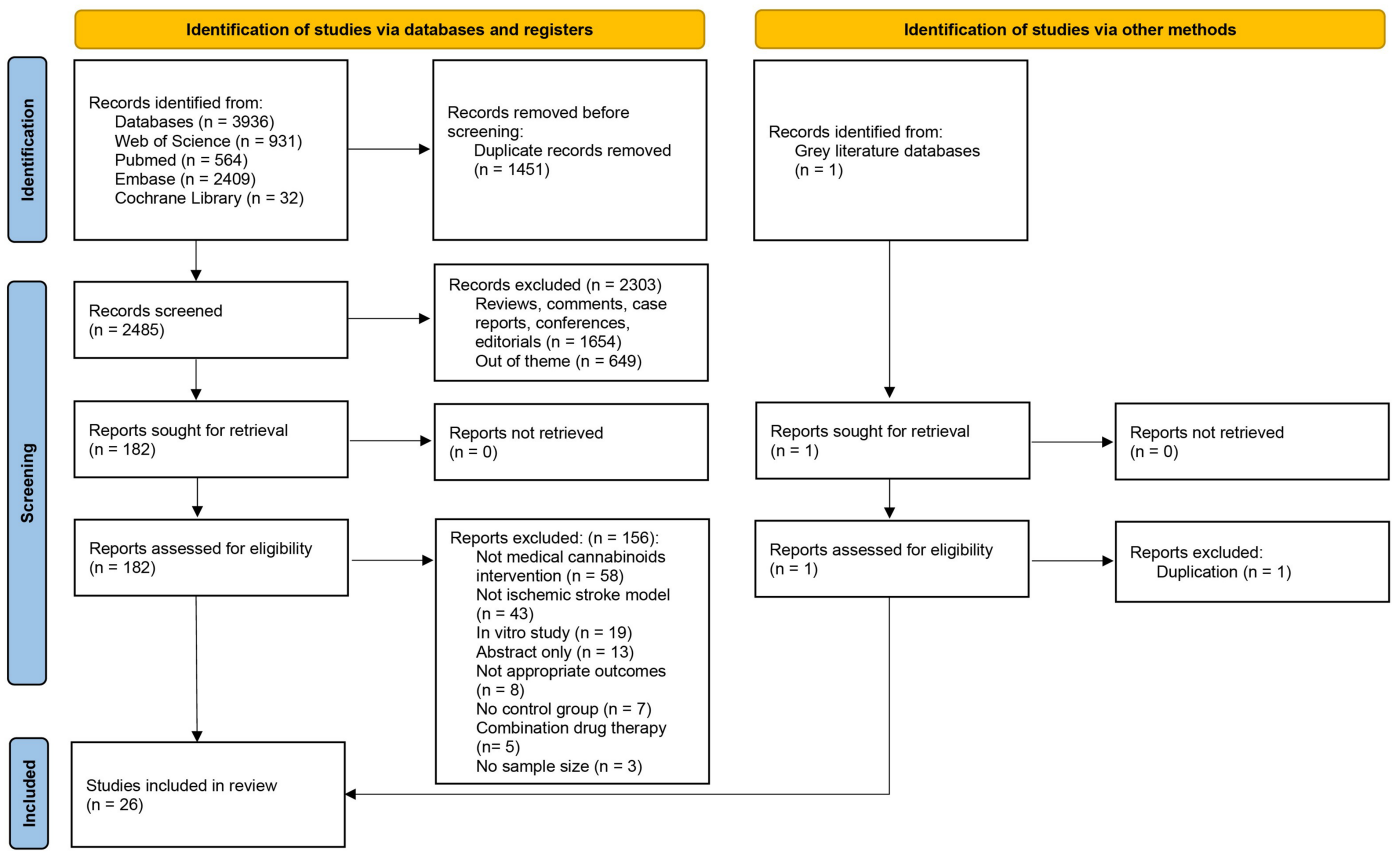
Study selection flowchart.

A systematic search of WoSCC, PubMed, Embase and Cochrane Library databases was conducted, and 3,936 records were initially retrieved. After removing duplicates, the remaining 2,485 records were screened by title and abstract, during which 2,303 were excluded as irrelevant to the research topic. Subsequently, full-text assessments were conducted on the 182 articles. Among these, 156 were excluded for the following reasons: use of non-target cannabinoids, non-ischemic stroke models, *in vitro* studies, unavailable full texts, insufficient outcome reporting, absence of a control group, concomitant use of other drugs, and lack of reported sample size. Additionally, one record from the grey literature was found to be already included in the above formally published databases. Ultimately, 26 articles met the predefined criteria and were included for the meta-analysis (Lavie et al., 2001; Leker et al., 2003; Teichner et al., 2003; Hayakawa et al., 2004; Mishima et al., 2005; Hayakawa et al., 2007a; Hayakawa et al., 2007b; Hayakawa et al., 2007c; Durmaz et al., 2008; Hayakawa et al., 2008; Hayakawa et al., 2009; Ceprián et al., 2017; Khaksar and Bigdeli, 2017a; Khaksar and Bigdeli, 2017b; Khaksar and Bigdeli, 2017c; Rodríguez-Muñoz et al., 2018; Yokubaitis et al., 2021; Khaksar et al., 2022; Liu et al., 2022; Meyer et al., 2022; Lavayen et al., 2023; Xu et al., 2023; Chen et al., 2024; Villa et al., 2024a; Villa et al., 2024b; de Souza Stork et al., 2025). The screening process was performed independently by two evaluators. The study selection process is illustrated in Figure 4.

The included studies spanned 2001 to June 2025. The experimental animals primarily consisted of rats and mice, with 15 and 11 studies using these species, respectively. Rat models comprised Wistar, Sprague–Dawley (SD), and spontaneously hypertensive rats (SHR), while mouse models included ddY, C57BL/6, and CD1 strains. The majority of experiments were conducted in male animals. Among the anesthesia methods used, isoflurane was the most frequently employed in 7 studies. Other anesthetics utilized included halothane, phenobarbital, sevoflurane, and ketamine-xylazine. Anesthetic details were not reported in 3 studies. 21 studies implemented a transient ischemia model, with ischemia durations ranging from 15 min to 4 h, while 5 studies utilized a permanent ischemia model. The MCs included CBD, THC, HU-211, VCE-004.8, HU-210, Abnormal cannabidiol (AB-CBD), and Full-spectrum *Cannabis sativa* extract (FSC), with CBD being the most extensively studied compound. Most studies employed intraperitoneal injection as the route of administration, followed by intraventricular and intravenous injections. One study utilized oral gavage. The timing of administration was categorized into 3 approaches: pre-ischemia, post-ischemia, or combined pre- and post-ischemia. In terms of outcome measures, 22 studies reported cerebral infarct volume. NFS were assessed in 11 studies, of which 4 studies used the modified Bederson score, 3 used the Bederson score, 2 used the Zea-Longa score, and 2 used the sensorimotor deficit score. Additional outcomes included CBF in 4 studies, apoptosis (TUNEL-positive cells) in 6 studies, brain water content in 2 studies, BBB permeability in 2 studies, inflammatory cytokines (TNF-*α* and IL-1β) in 4 studies, and metabolic ratios (Glu/NAA and Lac/NAA ratios) in 2 studies. The basic characteristics of the included studies are shown in Table 3.

**Table 3:** Basic characteristics of the included studies.

Study	Animal characteristics	Anesthetic agent	Model	Interventions		Route	Treatment point	Outcome measures	Major targeting pathophysiology
				Experimental group	Control group				
Villa et al. (2024a)	Wistar rats	Sevoflurane	tMCAO (3 h)	CBD, 5 mg/kg	Vehicle	Ip	Post-MCAO	Cerebral infarct volume, TUNEL-positive cells	Apoptosis
Chen et al. (2024)	Male SD rats (270–300 g)	Isoflurane	tMCAO (2 h)	CBD, 5 mg/kg	Vehicle	Ip	Post-MCAO	IL-1β, TNF-α, iNOS, Iba1	Neuroinflammation
Xu et al. (2023)	Male SD rats (260–300 g)	Isoflurane	tMCAO (2 h)	CBD, 5 mg/kg	Vehicle	Ip	Post-MCAO	Cerebral infarct volume, NFS	Oxidative stress
Lavayen et al. (2023)	Male C57BL/6 mice (28–32 g)	Isoflurane	tMCAO (30 min)	VCE-004.8, 20 mg/kg	Vehicle	Ip	Post-MCAO	Cerebral infarct volume, BBB permeability, IL-1β-mRNA, MMP-9	Neuroinflammation, BBB, oxidative stress
Meyer et al. (2022)	C57BL/6 mice	Isoflurane	tMCAO (15 min)	CBD, 10 mg/kg	Vehicle	Ip	Post-MCAO	NFS	Neuroinflammation
Khaksar et al. (2022)	Male Wistar rats (230–330 g)	Ketamine and xylazine	tMCAO (60 min)	CBD, 100 ng	Vehicle	Icv	Pre-MCAO	Cerebral infarct volume, SOD, CAT, MDA, Bax, Bcl-2, caspase-3	Oxidative stress, apoptosis
Yokubaitis et al. (2021)	Male C57BL/6 mice (19–24 g)	Ketamine and xylazine	pMCAO	CBD, 30 mg/kg	Vehicle	Ip	Pre- and post-MCAO	Cerebral infarct volume	Neuroinflammation
Rodríguez-Muñoz et al. (2018)	Male CD1 mice	Isoflurane	pMCAO	CBD, 10 nmol	Saline	Icv	Post-MCAO	Cerebral infarct volume	Excitotoxicity
Khaksar and Bigdeli (2017a)	Male Wistar rats (250–350 g)	NR	tMCAO (60 min)	CBD, 100 ng	Vehicle	Icv	Pre-MCAO	Cerebral infarct volume, NFS, brain water content, BBB permeability	Excitotoxicity, BBB
Khaksar and Bigdeli (2017b)	Male Wistar rats (250–350 g)	NR	tMCAO (60 min)	CBD, 100 ng	Vehicle	Icv	Pre-MCAO	Cerebral infarct volume, NFS, brain water content, BBB permeability, TNF-α, TNFR1, NF-кB	Neuroinflammation, BBB
Ceprián et al. (2017)	Wistar rats	Sevoflurane	tMCAO (3 h)	CBD, 5 mg/kg	Vehicle	Ip	Post-MCAO	Cerebral infarct volume, TUNEL-positive cells, Glu/NAA ratio, Lac/NAA ratio	Excitotoxicity, apoptosis, neuroinflammation
Villa et al. (2024b)	Wistar rats	Sevoflurane	tMCAO (3 h)	VCE-004.8, 5 mg/kg	Vehicle	Ip	Post-MCAO	Cerebral infarct volume, TUNEL-positive cells, TNF-α, Glu/NAA ratio, Lac/NAA ratio	Excitotoxicity, oxidative stress, neuroinflammation
Liu et al. (2022)	Male SD rats (240–250 g)	Phenobarbital	tMCAO (1.5 h)	CBD, 3.2 mg/kg	Saline	Iv	Post-MCAO	Cerebral infarct volume, NFS, ROS, IL-1β, TNF-α	Oxidative stress, neuroinflammation
Khaksar and Bigdeli (2017c)	Male Wistar rats (250–350 g)	NR	tMCAO (60 min)	CBD, 100 ng	Vehicle	Icv	Pre-MCAO	Cerebral infarct volume, NF-kB, TNFR1	Neuroinflammation
Hayakawa et al. (2009)	Male ddY mice (25–35 g)	Halothane	tMCAO (4 h)	CBD, 3 mg/kg	Vehicle	Ip	Post-MCAO	NFS, TUNEL-positive cells	Neuroinflammation, apoptosis
Hayakawa et al. (2008)	Male ddY mice (25–35 g)	Halothane	tMCAO (4 h)	CBD, 3 mg/kg	Vehicle	Ip	Pre- and post-MCAO	Cerebral infarct volume, NFS, TUNEL-positive cells	Neuroinflammation, apoptosis
Durmaz et al. (2008)	SD rats (280–330 g)	Ketamine and xylazine	pMCAO	HU-211, 5 mg/kg	Vehicle	Iv	Pre-MCAO	TUNEL-positive cells, NO, cathepsin B, cathepsin L	Apoptosis
Hayakawa et al. (2007a)	Male ddY mice (25–35 g)	Halothane	tMCAO (4 h)	CBD, 3 mg/kg; THC, 10 mg/kg	Vehicle	Ip	Pre- and post-MCAO	Cerebral infarct volume, CBF, glutamate	Neuroinflammation, CBF, excitotoxicity
Hayakawa et al. (2007b)	Male ddY mice (25–35 g)	Halothane	tMCAO (4 h)	THC, 10 mg/kg	Vehicle	Ip	Pre- and post-MCAO	Cerebral infarct volume	-
Hayakawa et al. (2007c)	Male ddY mice (25–35 g)	Halothane	tMCAO (4 h)	CBD, 3 mg/kg; THC, 10 mg/kg	Vehicle	Ip	Pre- and post-MCAO	Cerebral infarct volume, CBF	CBF
Mishima et al. (2005)	Male ddY mice (25–35 g)	Halothane	tMCAO (4 h)	CBD, 1 mg/kg; AB-CBD, 3 mg/kg	Vehicle	Ip	Pre- and post-MCAO	Cerebral infarct volume, CBF	CBF
Hayakawa et al. (2004)	Male ddY mice (25–35 g)	Halothane	tMCAO (4 h)	CBD, 3 mg/kg; THC, 10 mg/kg	Vehicle	Ip	Pre- and post-MCAO	Cerebral infarct volume	-
Teichner et al. (2003)	Male SHR rats	Phenobarbital	pMCAO	HU-211, 4.5 mg/kg	Vehicle	Iv	Post-MCAO	Cerebral infarct volume, NFS	-
Leker et al. (2003)	SD rats (300 g)	Isoflurane	pMCAO	HU-210, 45 μg/kg	Vehicle	Iv	Post-MCAO	Cerebral infarct volume, NFS, CBF	CBF
Lavie et al. (2001)	SHR rats	Phenobarbital	pMCAO	HU-211, 4.5 mg/kg	Vehicle	Iv	Post-MCAO	Cerebral infarct volume, NFS	-
de Souza Stork et al. (2025)	Male Wistar rats (250–300 g)	Isoflurane	tMCAO (60 min)	FSC, 15 mg/kg	Vehicle	Gavage	Post-MCAO	Cerebral infarct volume, NFS, MDA, CAT	Oxidative stress

SD rats, Sprague–Dawley rats; SHR rats, spontaneously hypertensive rats; CBD, cannabidiol; THC, D-9-tetrahydrocannabinol; AB-CBD, Abnormal cannabidiol; FSC, Full-spectrum *Cannabis sativa* extract; TUNEL, terminal deoxynucleotidyl transferase (TdT)-mediated dUTP-biotin nick end labeling; IL-1β, interleukin-1β; TNF-α, tumor necrosis factor-α; iNOS, inducible nitric oxide synthase; Iba1, ionized calcium binding adaptor molecule 1; BBB, blood–brain barrier; MMP-9, Matrix metalloproteinase-9; SOD, superoxide dismutase; CAT, catalase; MDA, malondialdehyde; Bax, Bcl-2-associated X protein; Bcl-2, B-cell lymphoma 2; TNFR1, tumour necrosis factor receptor 1; NF-кB, nuclear factor-κB; Glu/NAA, glutamate/N-acetylaspartate; Lac/NAA, lactate/N-acetylaspartate; ROS, reactive oxygen species; NO, nitric oxide; CBF, cerebral blood flow; NFS, neurological function score; ip, intraperitoneal injection; icv., intraventricular injection; iv, intravenous injection; tMCAO, transient middle cerebral artery occlusion; pMCAO, permanent middle cerebral artery occlusion; NR, no report; h, hour; d, day; min, minute.

#### 3.2.2 Quality assessment of included studies

**Figure 5:**
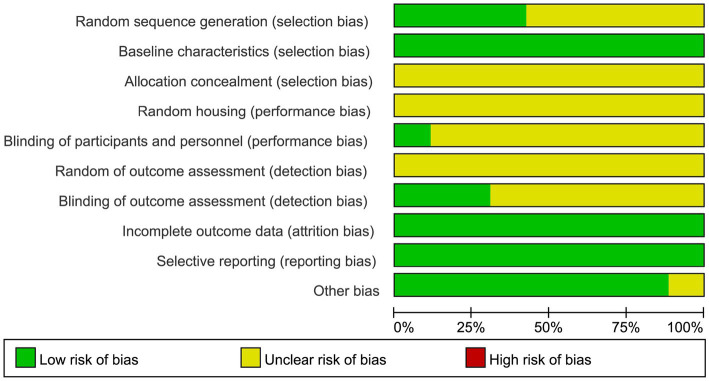
Risk of bias plot of the included studies.

Based on the SYRCLE risk of bias tool for animal studies, we evaluated the methodological quality of the 26 included studies. 11 studies employed a randomized controlled design. Specifically, one study used computer-generated random numbers, while 10 studies only mentioned randomization without providing detail. Baseline characteristics were well-balanced across all studies, with 3 studies reporting comparable gender ratios between groups. The methods for allocation concealment and random allocation to cages were not described in any study. Regarding blinding, 3 studies reported blinding of experimenters, and 8 reported blinding of outcome assessors. No study described methods for random outcome assessment. All studies had complete data with no evidence of selective reporting. 3 studies did not report specific anesthetics used, which might introduce potential biases. A summary of the risk of bias is presented in Figure 5. The detailed results of the quality assessment of included studies are shown in Supplementary Table 2 and Supplementary Figure 1.

#### 3.2.3 Cerebral infarct volume

**Figure 6:**
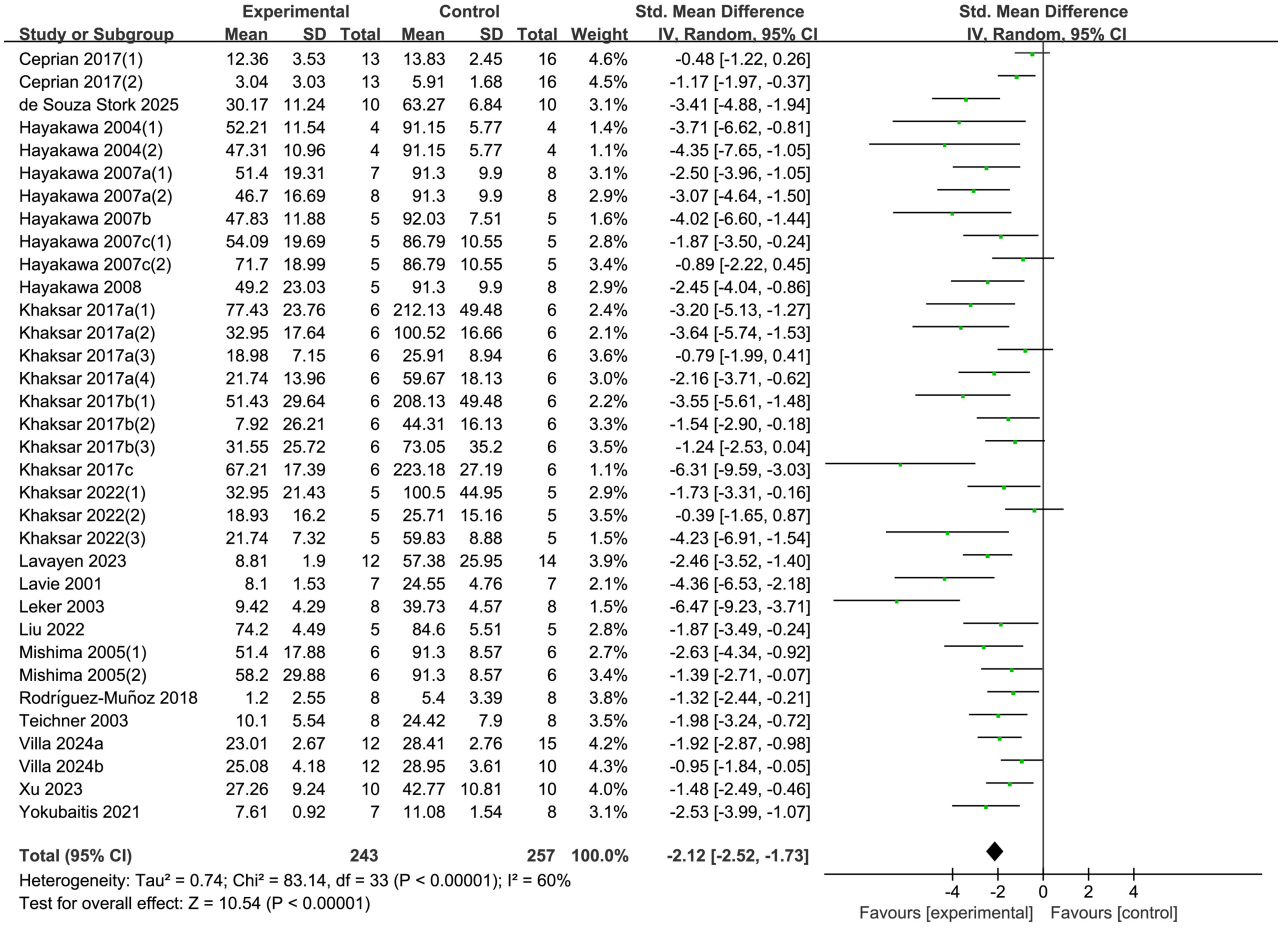
Forest plot of cerebral infarct volume.

22 studies reported changes in cerebral infarct volume after treatment. Heterogeneity across studies was significant (*p* < 0.00001, *I^2^* = 60%), necessitating the use of a random-effects model for analysis. The results demonstrated a significant reduction in cerebral infarct volume in the experimental group compared to the control group (SMD = −2.12, 95% CI [−2.52, −1.73], *p* < 0.00001; Figure 6).

#### 3.2.4 NFS

**Figure 7:**
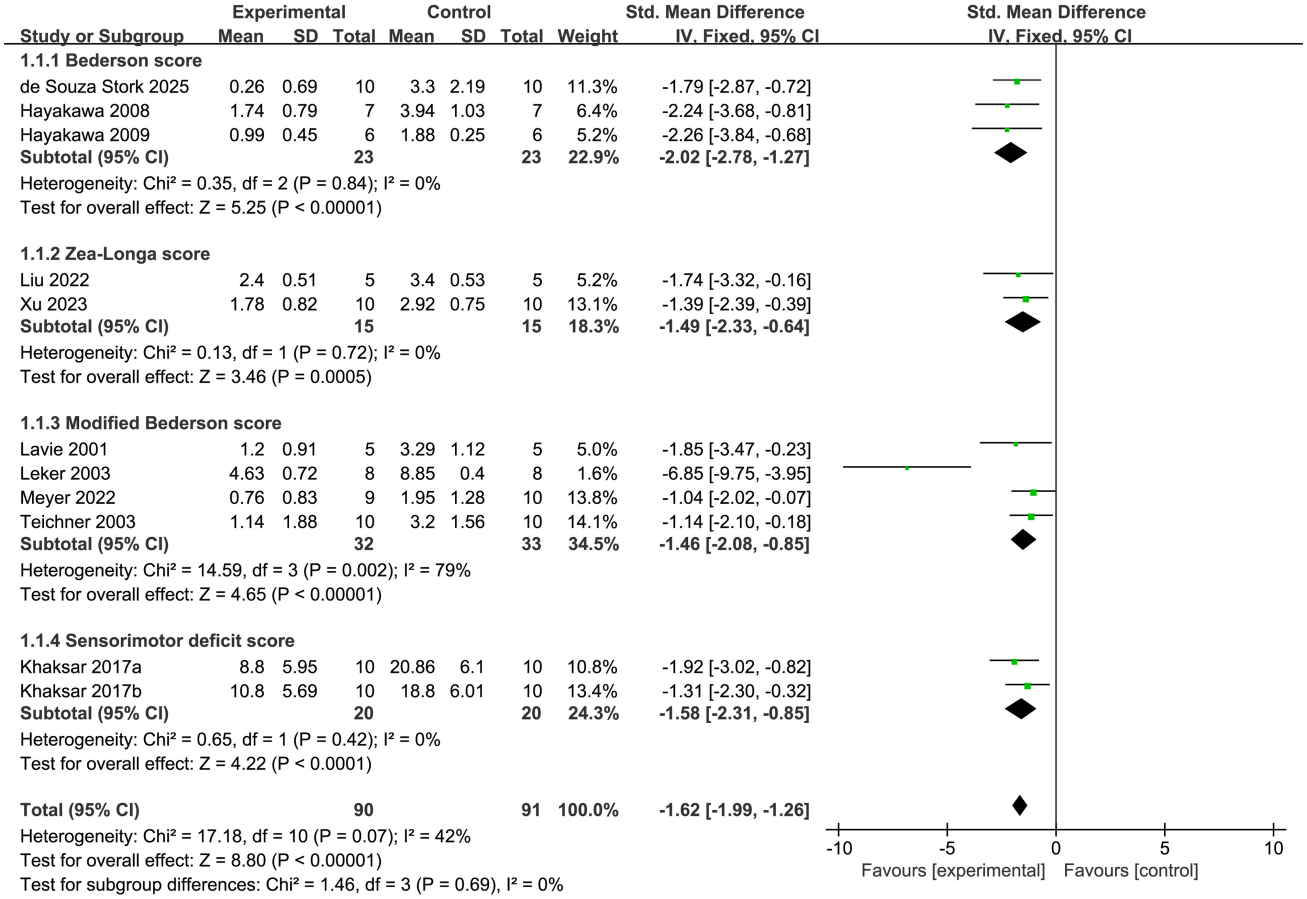
Forest plot of NFS.

11 studies assessed the impact of MCs on ischemic stroke using NFS. No significant heterogeneity was observed across the studies (*p* = 0.07, *I^2^* = 42%), prompting the use of a fixed-effects model for data analysis. The results showed that the improvement in NFS was significantly greater in the experimental group compared to the control group (SMD = −1.62, 95% CI [−1.99, −1.26], *p* < 0.00001; Figure 7). Notably, significant improvement trends were observed on multiple scoring scales, including the Bederson score (SMD = −2.02, 95% CI [−2.78, −1.27], *p* < 0.00001), Zea-Longa score (SMD = −1.49, 95% CI [−2.33, −0.64], *p* = 0.0005), modified Bederson score (SMD = −1.46, 95% CI [−2.08, −0.85], *p* < 0.00001), and sensorimotor deficit score(SMD = −1.58, 95% CI [−2.31, −0.85], *p* < 0.0001).

#### 3.2.5 CBF

**Figure 8:**
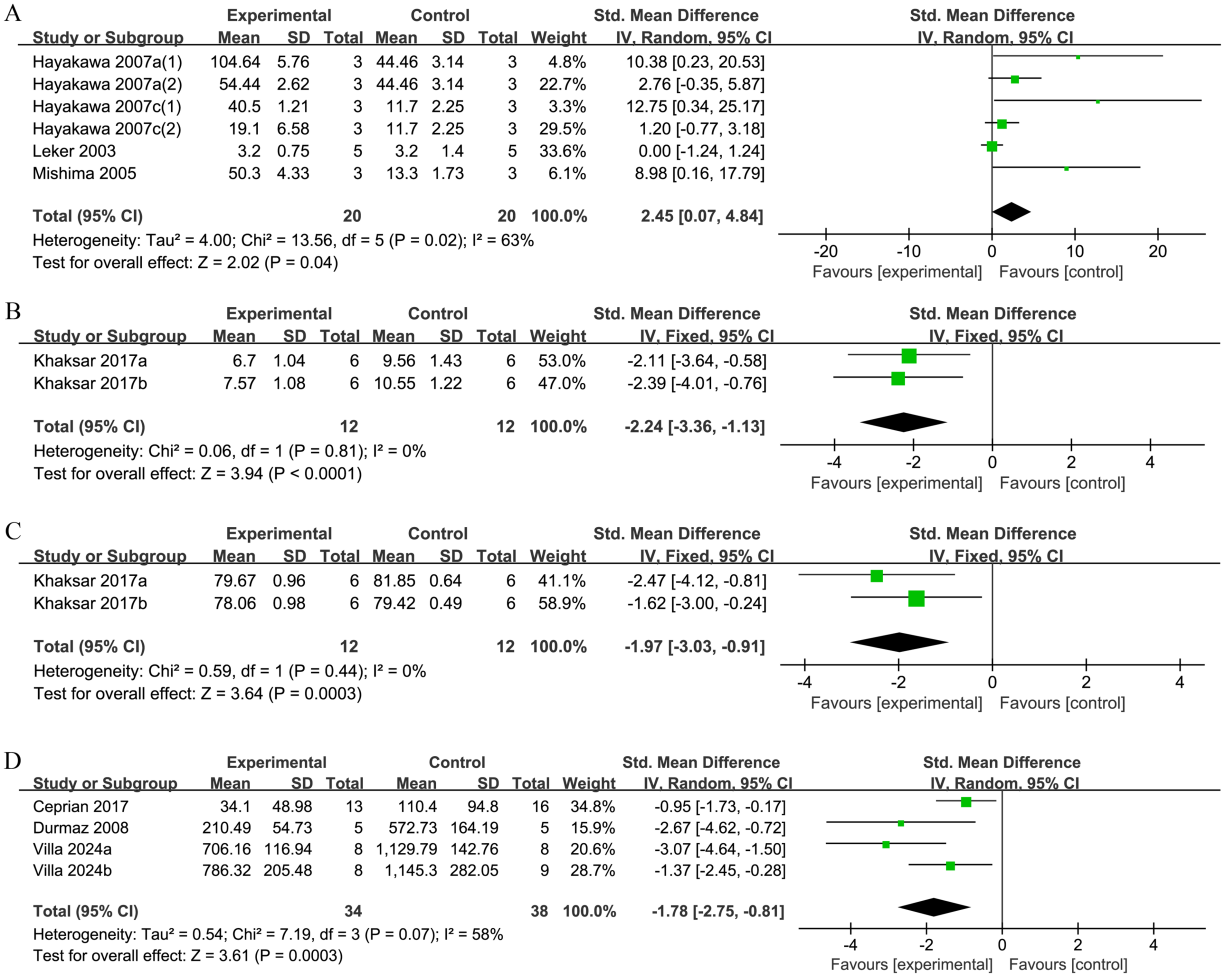
**(A)** Forest plot of CBF. **(B)** Forest plot of BBB permeability. **(C)** Forest plot of brain water content. **(D)** Forest plot of TUNEL-positive cells.

A total of 4 studies reported changes in CBF. Heterogeneity was detected among the studies (*p* = 0.02, *I^2^* = 63%), and a random-effects model was selected. The results demonstrated that compared to the control group, the experimental group showed a significant enhancement in the recovery of CBF after ischemic injury (SMD = 2.45, 95% CI [0.07, 4.84], *p* = 0.04; Figure 8A).

#### 3.2.6 BBB permeability

In total, 2 studies reported on BBB permeability indicators. There was no significant heterogeneity among these studies (*p* = 0.81, *I^2^* = 0%), leading to the selection of a fixed-effects model. The results showed that the experimental group effectively reduced BBB permeability (SMD = −2.24, 95% CI [−3.36, −1.13], *p* < 0.0001; Figure 8B).

#### 3.2.7 Brain water content

A total of 2 studies examined brain water content. No heterogeneity was observed across studies (*p* = 0.44, *I^2^* = 0%), thus a fixed-effects model was selected. The results demonstrated that the brain water content of the experimental group was significantly reduced after treatment (SMD = −1.97, 95% CI [−3.03, −0.91], *p* = 0.0003; Figure 8C).

#### 3.2.8 TUNEL-positive cells

A total of 4 studies reported changes in the number of TUNEL-positive cells. Heterogeneity was detected among the studies (*p* = 0.07, *I^2^* = 58%), thus a random-effects model was used for analysis. The analysis showed that the number of TUNEL-positive cells in the experimental group was significantly lower than that in the control group (SMD = −1.78, 95% CI [−2.75, −0.81], *p* = 0.0003; Figure 8D).

#### 3.2.9 Oxidative stress markers

One study reported that administration of CBD in rats enhanced the activity of superoxide dismutase (SOD) (SMD = 4.02, 95% CI [1.44, 6.60], *p* = 0.002; Supplementary Figure 2A) and catalase (CAT) (SMD = 2.48, 95% CI [0.61, 4.35], *p* = 0.009; Supplementary Figure 2B) in brain tissue, while reducing malondialdehyde (MDA) levels (SMD = −4.59, 95% CI [−7.46, −1.73], *p* = 0.002; Supplementary Figure 2C). Another study reported that CBD treatment significantly decreased reactive oxygen species (ROS) levels in the brain (SMD = −1.67, 95% CI [−2.77, −0.56], *p* = 0.003; Supplementary Figure 2D). In another study, FSC treatment increased the activity of CAT in lung tissue (SMD = 5.26, 95% CI [3.24, 7.27], *p* < 0.00001; Supplementary Figure 2E), and reduced MDA levels (SMD = −2.01, 95% CI [−3.12, −0.89], *p* = 0.0004; Supplementary Figure 2F).

#### 3.2.10 TNF-α level

**Figure 9:**
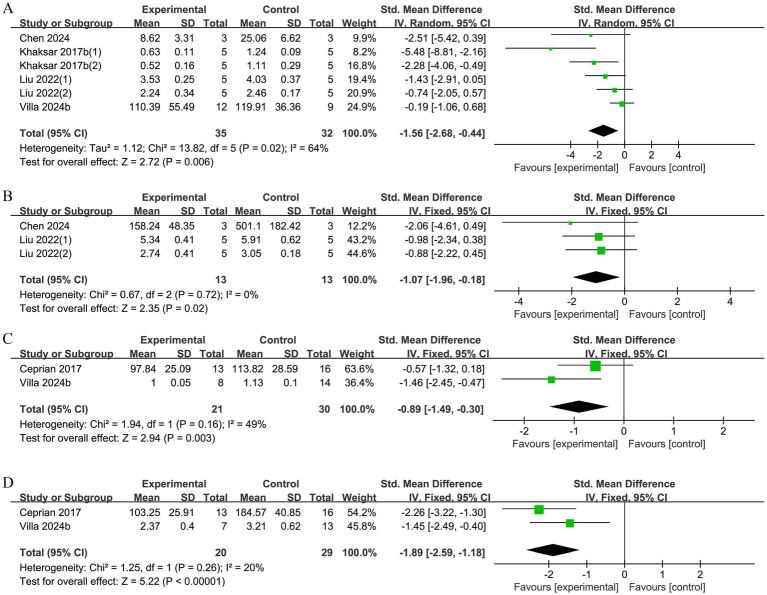
**(A)** Forest plot of TNF-α level. **(B)** Forest plot of IL-1β level. **(C)** Forest plot of Glu/NAA ratio. **(D)** Forest plot of Lac/NAA ratio.

A total of 4 research reports examined TNF-α levels, with significant heterogeneity detected among the studies (*p* = 0.02, *I^2^* = 64%). Consequently, a random-effects model was selected for further analysis. The results showed that the experimental group effectively reduced the content of TNF-α (SMD = −1.56, 95% CI [−2.68, −0.44], *p* = 0.006, Figure 9A).

#### 3.2.11 IL-1β level

A total of 2 studies reported on IL-1β levels. There was no heterogeneity across studies (*p* = 0.72, *I^2^* = 0%), and a fixed-effects model was chosen for analysis. The results showed that the experimental group effectively reduced the level of IL-1β (SMD = −1.07, 95% CI [−1.96, −0.18], *p* = 0.02; Figure 9B).

#### 3.2.12 Glu/NAA ratio

A total of 2 studies examined the Glu/NAA ratio. There was no heterogeneity among the studies (*p* = 0.16, *I^2^* = 49%), so the analysis was performed using a fixed-effects model. The results showed that the experimental group significantly improved the Glu/NAA ratio (SMD = −0.89, 95% CI [−1.49, −0.30], *p* = 0.003; Figure 9C).

#### 3.2.13 Lac/NAA ratio

In total, 2 studies measured the Lac/NAA ratio. There was no heterogeneity among these studies (*p* = 0.26, *I^2^* = 20%), so a fixed-effects model was selected. The results showed that the Lac/NAA ratio was significantly decreased in the experimental group (SMD = −1.89, 95% CI [−2.59, −1.18], *p* < 0.00001; Figure 9D).

#### 3.2.14 Subgroup analysis

To explore potential sources of heterogeneity, we performed subgroup analyses of cerebral infarct volume, CBF, TUNEL and TNF-*α* according to animal species, anesthetic agent, model type, occlusion duration, drug class, route of administration and timing of administration.

For cerebral infarct volume, no significant subgroup heterogeneity was found across animal species (*p* = 0.45), model type (*p* = 0.20), route of administration (*p* = 0.11), or timing of administration (*p* = 0.31). Significant differences in subgroup effect sizes were detected for anesthetic agent (*p* = 0.03), occlusion duration (*p* = 0.03) and drug class (*p* = 0.01). In terms of drug class, various MCs showed a positive effect on reducing infarct volume. Among them, CBD exhibited the most robust therapeutic effect (SMD = −1.89, 95% CI [−2.31, −1.46]), supported by 23 comparative analyses. Among anesthetics, isoflurane demonstrated the largest effect size (SMD = −2.61, 95% CI [−3.80, −1.42]). For occlusion duration, permanent occlusion (SMD = −2.96, 95% CI [−4.36, −1.55]) exhibited the largest effect size in reducing infarct volume. Among the transient occlusion groups, a 60-min occlusion (SMD = -2.33, 95% CI [−3.13, −1.52]) yielded more consistent pooled results. Furthermore, infarct volume reductions were consistently observed in both transient and permanent MCAO models. Continuous administration (SMD = −2.56, 95% CI [−3.13, −1.98]) was more effective than pre-MCAO (SMD = −2.02, 95% CI [−2.72, −1.32]) or post-MCAO (SMD = −1.95, 95% CI [−2.57, −1.32]) administration. The results of the subgroup analysis of cerebral infarct volume are shown in Table 4.

**Table 4:** Subgroup analysis of cerebral infarct volume.

Subgroup	Study	SMD (95% CI)	Heterogeneity *I^2^* (%)	*p* value	Subgroup (*p* value)
Animal species					0.45
CD1 mice	1	−1.32 (−2.44, −0.21)	-	-	
C57BL/6 mice	2	−2.48 (−3.34, −1.62)	0	0.94	
DdY mice	10	−2.29 (−2.91, −1.67)	22	0.24	
SD rats	3	−2.92 (−5.20, −0.63)	82	0.004	
SHR rats	2	−3.00 (−5.30, −0.70)	71	0.06	
Wistar rats	16	−1.87 (−2.45, −1.29)	66	< 0.0001	
Anesthetic agent					0.03
Halothane	10	−2.29 (−2.91, −1.67)	22	0.24	
Isoflurane	5	−2.61 (−3.80, −1.42)	75	0.003	
Ketamine and xylazine	4	−1.95 (−3.36, −0.55)	66	0.03	
Phenobarbital	3	−2.52 (−3.85, −1.19)	49	0.14	
Sevoflurane	4	−1.09 (−1.67, −0.51)	48	0.13	
Other	8	−2.39 (−3.36, −1.42)	62	0.01	
Occlusion duration					0.03
30 min	1	−2.46 (−3.52, −1.40)	-	-	
60 min	12	−2.33 (−3.13, −1.52)	64	0.001	
1.5 h	1	−1.87 (−3.49, −0.24)	-	-	
2 h	1	−1.48 (−2.49, −0.46)	-	-	
3 h	4	−1.09 (−1.67, −0.51)	48	0.13	
4 h	10	−2.29 (−2.91, −1.67)	22	0.24	
Permanent occlusion	5	−2.96 (−4.36, −1.55)	74	0.004	
Model type					0.20
PMCAO	5	−2.96 (−4.36, −1.55)	74	0.004	
TMCAO	29	−1.99 (−2.40, −1.59)	57	0.0001	
Drug class					0.01
AB-CBD	1	−1.39 (−2.71, −0.07)	-	-	
CBD	23	−1.89 (−2.31, −1.46)	52	0.002	
FSC	1	−3.41 (−4.88, −1.94)	-	-	
HU-210	1	−6.47 (−9.23, −3.71)	-	-	
HU-211	2	−3.00 (−5.30, −0.70)	71	0.06	
THC	4	−2.76 (−4.44, −1.08)	64	0.04	
VCE-004.8	2	−1.67 (−3.15, −0.19)	78	0.03	
Route of administration					0.11
Gavage	1	−3.41 (−4.88, −1.94)	-		
Icv	12	−2.07 (−2.81, −1.34)	60	0.004	
Ip	17	−1.85 (−2.31, −1.40)	52	0.007	
Iv	4	−3.38 (−5.20, −1.55)	74	0.009	
Timing of administration					0.31
Pre-MCAO	13	−2.02 (−2.72, −1.32)	58	0.005	
Post-MCAO	12	−1.95 (−2.57, −1.32)	72	< 0.0001	
Pre- and post-MCAO	9	−2.56 (−3.13, −1.98)	0	0.60	

SD rats, Sprague–Dawley rats; SHR rats, spontaneously hypertensive rats; CBD, cannabidiol; THC, D-9-tetrahydrocannabinol; AB-CBD, Abnormal cannabidiol; FSC, Full-spectrum *Cannabis sativa* extract; ip, intraperitoneal injection; icv., intraventricular injection; iv, intravenous injection; tMCAO, transient middle cerebral artery occlusion; pMCAO, permanent middle cerebral artery occlusion.

For CBF, drug class (*p* = 0.002) and route of administration (*p* = 0.02) were the primary influencing factors. CBD significantly increased CBF (SMD = 10.29, 95% CI [4.42, 16.15]), an effect substantially greater than that observed with THC (SMD = 1.65, 95% CI [−0.02, 3.32]). Intraperitoneal injection (SMD = 4.13, 95% CI [0.77, 7.50]) was superior to intravenous administration (SMD = 0.00, 95% CI [−1.24, 1.24]). No significant heterogeneity was found for timing of administration (*p* = 0.07). Continuous administration showed a trend toward greater CBF improvement (SMD = 5.62, 95% CI [0.54, 10.70]). The results of the subgroup analysis of CBF are shown in Supplementary Table 3.

For TUNEL, no significant subgroup differences were found across stratified factors (*p* = 0.51, *p* = 0.35, *p* = 0.35). Nevertheless, two cannabinoids, HU-211 (SMD = −2.67, 95% CI [−4.62, −0.72]) and VCE-004.8 (SMD = −1.37, 95% CI [−2.45, −0.28]), showed potential protective effects. The results of the subgroup analysis of TUNEL are shown in Supplementary Table 4.

For TNF-*α*, no significant subgroup differences were observed for animal species (*p* = 0.46), anesthetic agent (*p* = 0.09), route of administration (*p* = 0.30) or timing of administration (*p* = 0.08). Significant heterogeneity in subgroup effect sizes was identified across drug class (*p* = 0.02). Among them, CBD administration demonstrated the largest effect size for reducing TNF-α levels (SMD = −1.97, 95% CI [−3.18, −0.75]). The results of the subgroup analysis of TNF-α are shown in Supplementary Table 5.

#### 3.2.15 Publication bias

**Figure 10:**
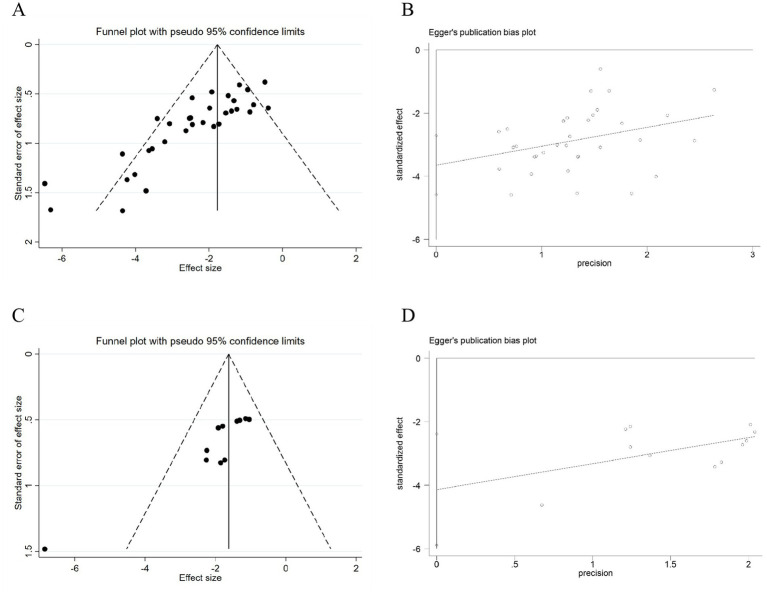
**(A)** Funnel plot of cerebral infarct volume. **(B)** Egger’s plot of cerebral infarct volume. **(C)** Funnel plot of NFS. **(D)** Egger’s plot of NFS.

In this study, publication bias was assessed for two outcome indicators, including infarct volume and NFS (Figure 10). The analysis revealed that the funnel plots for both indicators showed asymmetric distribution characteristics. The Egger test further indicated significant publication bias for infarct volume (*p* < 0.001) and NFS (*p* < 0.001). A trim-and-fill analysis was subsequently performed to evaluate the potential impact of publication bias (Supplementary Figure 3). The results showed a minimal deviation between the adjusted and original effect sizes. This suggests that despite the presence of publication bias, the results of this study remain robust, as such bias is insufficient to materially change the overall conclusions.

## 4 Discussion

### 4.1 Summary of evidence

In recent years, the advancement of cannabis legalization has sparked increasing interest among researchers regarding the potential therapeutic effects of MCs in ischemic cerebrovascular diseases. We conducted a bibliometric mapping of MCs research in cerebral ischemia over the past 25 years. The results indicated that the United States emerged as the most prolific contributor to this field. However, the visual networks revealed a tendency for researchers to collaborate primarily within their own countries and institutions, highlighting a need for cross-national, inter-agency and inter-team communication and collaboration. Notably, this study integrated diverse models of cerebral ischemia at both cellular and *in vivo* levels. This broad model selection not only enhances the representativeness of the study’s conclusions for the entire field of cerebral ischemia but also provides a more comprehensive perspective on the field’s core focus and evolution. Based on this, the study identified universal and robust research hotspots in cerebral ischemia, primarily including the neuroprotective mechanisms of MCs, the exploration of experimental models, and the multitarget effects of CBD and THC.

Our meta-analysis section focused on studies of focal cerebral ischemia utilizing the MCAO model. The results showed that MCs significantly improved infarct volume, NFS, CBF, BBB permeability, brain water content, oxidative stress markers, TNF-α levels, IL-1β levels, TUNEL-positive cells, Glu/NAA ratio, and Lac/NAA ratio in animal models. These findings suggest that MCs can effectively alleviate acute ischemia-mediated brain injury and exert neuroprotective effects.

### 4.2 Potential mechanisms of MCs on ischemic stroke

**Figure 11:**
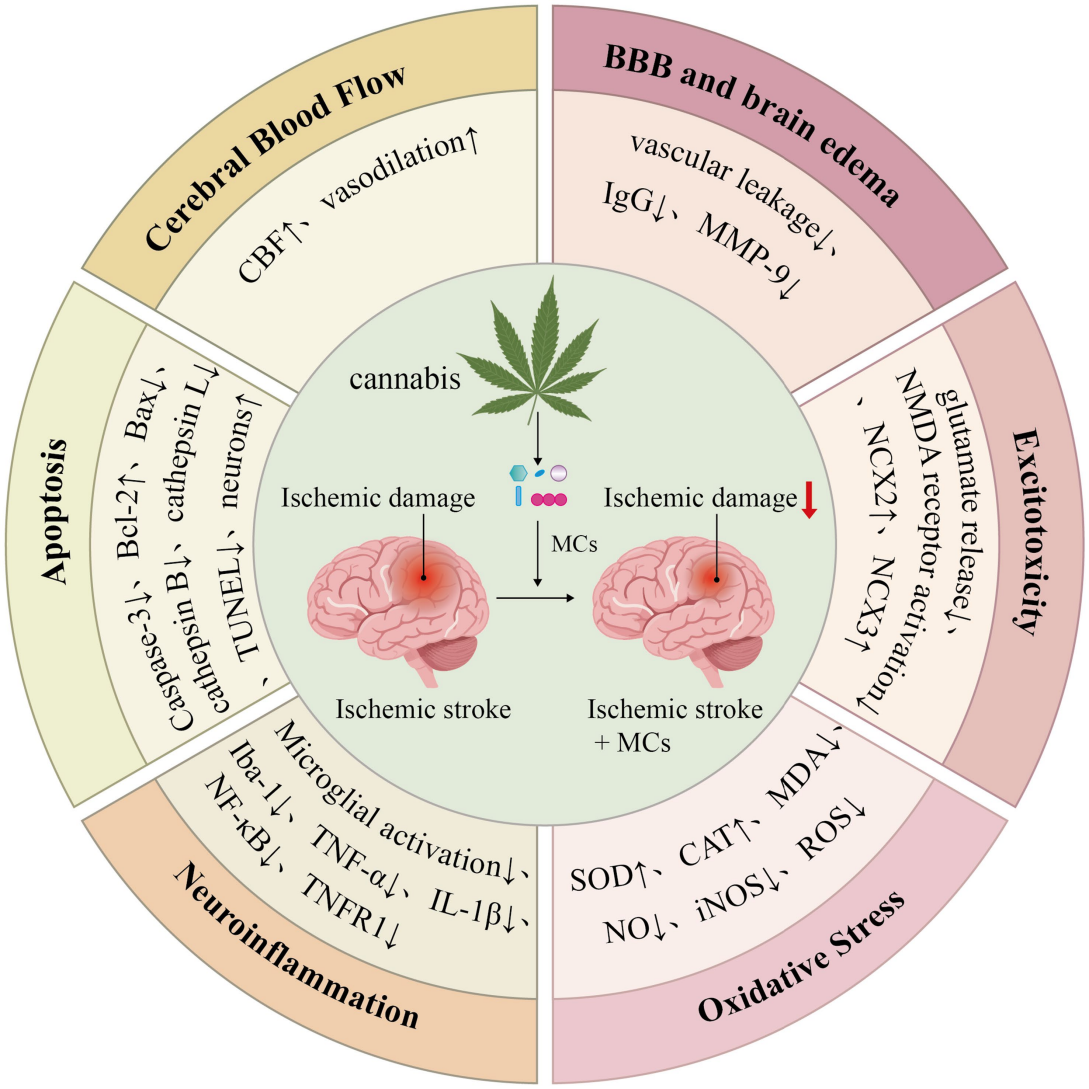
Neuroprotective mechanisms of MCs in ischemic stroke. MCs, medical cannabinoids; CBF, cerebral blood flow; BBB, blood–brain barrier; IgG, immunoglobulin G; MMP-9, Matrix metalloproteinase-9; NMDA receptor, N-methyl-d-aspartate receptor; NCX2, Na^+^/Ca^2+^ exchanger 2; NCX3, Na^+^/Ca^2+^ exchanger 3; SOD, superoxide dismutase; CAT, catalase; MDA, malondialdehyde; NO, nitric oxide; iNOS, inducible nitric oxide synthase; ROS, reactive oxygen species; Iba-1, ionized calcium binding adaptor molecule 1; IL-1β, interleukin-1β; TNF-α, tumor necrosis factor-α; TNFR1, tumour necrosis factor receptor 1; NF-кB, nuclear factor-κB; Bcl-2, B-cell lymphoma 2; Bax, Bcl-2-associated X protein; TUNEL, terminal deoxynucleotidyl transferase (TdT)-mediated dUTP-biotin nick end labeling.

Integrating the bibliometric insights with the findings of our meta-analysis, we comprehensively summarized the potential neuroprotective mechanisms of MCs in ischemic stroke. These mechanisms primarily include the regulation of CBF, preservation of BBB integrity and reduction of associated cerebral edema, as well as modulation of oxidative stress, excitotoxicity, neuroinflammation, and apoptosis (Figure 11).

#### 4.2.1 CBF

MCs can enhance CBF in ischemic brain tissue by modulating cerebral vascular tone (Benyó et al., 2016; Richter et al., 2018). In a mouse model of focal cerebral ischemia, the reduction of CBF in the infarction site was reversed by treatment with 3.0 mg/kg CBD through activation of the 5-HT1A receptor (Mishima et al., 2005). Additionally, THC can facilitate redistribution of blood flow in ischemic brain regions, with acute administration increasing CBF in specific areas such as the anterior cingulate cortex, frontal cortex, and insula (Ogunbiyi et al., 2020). One study further demonstrated that both CBD and THC can significantly increase cortical blood flow when administered via intraperitoneal injection; moreover, the efficacy of CBD remained stable after repeated dosing for 14 days (Hayakawa et al., 2007c). Therefore, MCs can protect the animal brain from ischemic injury by improving perfusion.

#### 4.2.2 BBB and cerebral edema

The breakdown of BBB is a critical pathological feature following ischemic stroke (Qiu et al., 2021). The primary mechanisms involve the destruction of tight junction proteins and enhanced vesicular transport, consequently leading to leakage of peripheral neurotoxic substances and cerebral edema (Candelario-Jalil et al., 2022; Zhu et al., 2024). CBD has been shown to significantly reduce Evans Blue extravasation and the degree of cerebral edema in the ischemic hemisphere of MCAO rats (Khaksar and Bigdeli, 2017a). In the OGD model, CBD attenuated cellular permeability by activating the peroxisome proliferator-activated receptor *γ* (PPAR-γ) and 5-HT1A signaling pathways (Hind et al., 2016). In addition, a key mechanism underlying BBB disruption is the upregulation of matrix metalloproteinase-9 (MMP-9), which degrades extracellular matrix and tight junction proteins (Ji et al., 2023). CBD amino quinone derivatives have been found to reduce BBB leakage in stroke mice, potentially through inhibiting MMP-9 expression in brain tissue (Lavayen et al., 2023).

#### 4.2.3 Oxidative stress

Following ischemic stroke, the impaired energy metabolism in brain tissue leads to overproduction of ROS and reactive nitrogen species (RNS), thereby inducing oxidative stress injury (Li et al., 2022). Experimental evidence suggested that CBD could modulate the content of ROS by targeting mitofusin-2 (MFN2) protein (Xu et al., 2023). The overactivation of inducible nitric oxide synthase (iNOS) sharply raises intracellular RNS levels (Wu et al., 2023). Administration of CBD significantly reduced post-ischemic ROS and iNOS levels, potentially mediated by regulating the activity of cyclin-dependent kinase regulatory subunit 1B (CKS1B) (Chen et al., 2024). SOD and CAT are key endogenous antioxidant enzymes that can alleviate neurotoxicity by scavenging oxygen free radicals (Afzal et al., 2023). Additionally, the overload of free radicals induces lipid peroxidation in the cell membrane, resulting in the accumulation of damaging markers like MDA (Pawluk et al., 2024). CBD enhanced SOD and CAT activity, and reduced MDA levels in ischemic lesion sites (Khaksar et al., 2022). FSC has also been demonstrated to have antioxidant effects by modulating peripheral organ oxidative stress parameters, such as MDA, CAT, and nitric oxide (NO) levels (de Souza Stork et al., 2025). Additionally, HU-210 effectively inhibited the overproduction of ROS within mitochondria, thereby enhancing the tolerance of neural tissue to ischemic injury (Cai et al., 2017).

#### 4.2.4 Excitotoxicity

The initial ischemic event leads to neuronal ATP depletion and ion channel dysfunction, subsequently causing cell membrane depolarization and massive influx of Ca^2+^. This process promotes the exocytosis of glutamate vesicles (Li et al., 2026). Excess glutamate activates both ionotropic and metabotropic glutamate receptors on the postsynaptic membrane, thereby inducing excitotoxic injury (Yang et al., 2024). CBD can inhibit the excitatory effects following ischemic stroke, which is associated with its reduction of Glu/NAA levels in brain tissue (Ceprián et al., 2017). Its derivative, VCE-004.8, also exhibits similar neuroprotective effects (Villa et al., 2024b). Another study demonstrated that THC suppressed glutamate release from presynaptic terminals of hippocampal neurons, thereby attenuating synapse-mediated neuronal damage (Gilbert et al., 2007). Moreover, CBD and THC have been shown to effectively reduce neurotoxicity mediated by N-methyl-d-aspartate (NMDA) and 2-amino-3-(4-butyl-3-hydroxyisoxazol-5-yl)propionic acid (AMPA) receptors (Hampson et al., 1998). HU-211 can directly inhibit the activity of NMDA receptor (Fernández-Ruiz et al., 2015). Excitotoxicity-induced disruption of intracellular calcium homeostasis is a critical pathogenic mechanism in ischemic brain injury. CBD counteracted Ca^2+^ overload-induced neurotoxicity by upregulating the expression of Na+/Ca2 + exchanger 2 (NCX2) and Na+/Ca2 + exchanger 3 (NCX3) in cortical neurons (Khaksar and Bigdeli, 2017a).

#### 4.2.5 Neuroinflammation

Acute CBF interruption-induced neuroinflammation is a critical pathogenesis of ischemic stroke, typically leading to poor prognosis. The inflammatory response initiates within minutes of cerebral ischemia and persists throughout all stages of the disease (Alsbrook et al., 2023). Microglia serve as key effector cells that trigger post-stroke neuroinflammation (Endres et al., 2022). Ischemic injury rapidly activates microglia, leading to the substantial release of pro-inflammatory factors such as TNF-*α* and IL-1β, thereby triggering an inflammatory cascade (Kumari et al., 2024). These cytokines also activate the nuclear factor-κB (NF-κB) pathway, inducing the production of more inflammatory mediators and exacerbating neurotoxicity (Mussbacher et al., 2023). The expression levels of TNF-α and IL-1β in brain tissue are closely related to the degree of nerve injury (Tirandi et al., 2023). In the MCAO models, the mRNA and protein expression levels of both TNF-α and IL-1β are significantly upregulated (DeLong et al., 2022; Xu et al., 2024). MCs such as CBD (Henshaw et al., 2021), VCE-004.8 (Navarrete et al., 2022), and FSC (Maayah et al., 2020) can reduce the levels of these two mediators, which highlights their potential in mitigating neuroinflammation.

#### 4.2.6 Apoptosis

The neuronal apoptosis in the penumbra is a pivotal mechanism underlying the progression of brain injury after ischemic stroke. The intrinsic apoptotic pathway is centrally regulated by the B-cell lymphoma 2 (Bcl-2) family, which includes pro-apoptotic members [e.g., Bcl-2-associated X protein (Bax), Bcl-xL/Bcl-2-associated death promoter (Bad)] and anti-apoptotic factors [e.g., Bcl-2, B-cell lymphoma-extra-large (Bcl-xL)] (Uzdensky, 2019). Under ischemic conditions, the balance shifts toward pro-apoptotic signaling in neurons. This process ultimately manifests in characteristic pathological features at the cellular level, such as DNA degradation, nuclear condensation, organelle destruction, and apoptotic body formation (Vitale et al., 2023). MCs have demonstrated anti-apoptotic effects. CBD not only reduced caspase-3 activity and the ratio of apoptotic bodies in hippocampal neurons but also inhibited the apoptotic pathway by modulating the Bax/Bcl-2 balance (Sun et al., 2017; Khaksar et al., 2022). HU-210 was shown to rescue neuronal apoptosis and limit expansion of the lesion in a rat MCAO model (Cai et al., 2017). HU-211 can affect both caspase-dependent and -independent apoptotic processes by reducing the activity of cathepsin B and cathepsin L (Durmaz et al., 2008; Yagami et al., 2019). Our study demonstrated that MCs reduced the number of TUNEL-positive cells after cerebral ischemia.

### 4.3 Interpretation of subgroup analysis results

A multivariable subgroup analysis was conducted to examine the effects of different conditions on cerebral infarct volume, CBF, TUNEL-positive cells, and TNF-*α* levels. The animal species was not a significant source of heterogeneity. Despite inter-individual differences, the overall trend suggested that the interventional effect on infarct volume was consistent across species, with Wistar rats being the most frequently employed rodent model.

Several anesthetics influenced both cerebral infarct volume and the observed heterogeneity. Among the anesthetics evaluated (e.g., halothane, isoflurane, phenobarbital), isoflurane was associated with the largest pooled effect size. Although isoflurane may confer a degree of neuroprotection during cerebral ischemia, evidence suggests that it does not alter the overall pathological progression of ischemic stroke (Scheid et al., 2023). This indicates that its use has no major confounding effect (Sarraf-Yazdi et al., 1998; Janssen et al., 2004). Additionally, the advantages of isoflurane in animal models are noteworthy (Maud et al., 2014). For instance, it is easy to operate, takes effect rapidly, delivers stable effects, and has a favorable safety profile (Oh and Narver, 2024). These characteristics also meet the standards for animal welfare and research ethics. Given its pharmacological profile and widespread utility, inhaled isoflurane represents an efficient and appropriate anesthetic choice when establishing MCAO models (Kitano et al., 2007; Hillman et al., 2019; Shi et al., 2020).

Differences in occlusion durations might be a source of heterogeneity. The results of this study indicated that permanent occlusion and 60-min transient occlusion offered certain advantages in clarifying the impact of MCs on cerebral infarct volume. This is possibly attributable to the relatively typical degree of ischemic brain injury and pathophysiological response under these two conditions. Regardless of transient or permanent occlusion, the evolution of the infarct area usually follows a progressive pattern from the striatum to the cerebral cortex (Fluri et al., 2015). In addition, one study showed that sustained blood flow obstruction for 60 to 120 min can induce ischemic hemispheric necrosis similar to that seen in human stroke (Sommer, 2017). It is necessary to further clarify the optimal duration of ischemia in future stroke studies.

MCs significantly reduced cerebral infarct volume in both transient and permanent MCAO models, and model type was not a source of heterogeneity. The pMCAO model demonstrated a larger effect size, whereas the tMCAO model exhibited more consistent results across studies. Therefore, future research should include more comparisons to determine which model more accurately recapitulates the clinical course of cerebral ischemia.

The subgroup analysis by drug class demonstrated CBD’s advantages in improving cerebral infarct volume, CBF, and TNF-α levels. These findings are consistent with its known mechanisms of anti-inflammatory effects, microcirculatory improvement, and neuroprotection (Martinez Naya et al., 2023; Singh et al., 2023). Additionally, CBD was effective in reducing NFS, as shown in Supplementary Figure 4. It is noteworthy that the effect size of CBD in increasing CBF may be overestimated (SMD = 10.29). This is likely due to the high standardization of preclinical studies (smaller within-group variation and larger between-group differences) and methodological limitations (lack of blinding and small sample sizes). Therefore, its preclinical and clinical value in CBF requires further clarification. Similarly, THC decreased infarct volume in stroke models, although the evidence is limited by the small number of comparisons. The effect of THC on CBF exhibited a modest upward trend that did not reach statistical significance, warranting further investigation. Nevertheless, one study reported that acute THC administration increased CBF in anesthetized stroke animals, whereas chronic dosing elevated peripheral arterial flow instead (Sultan et al., 2018). HU-210 demonstrated the largest effect size in reducing infarct volume, but this finding was based on a single study, potentially overestimating its efficacy. Additionally, the single-target action of HU-210 could limit its biological efficacy within complex organisms, particularly in the regulation of CBF. HU-211 and VCE-004.8 also showed modest benefits on infarct volume and TUNEL staining. Collectively, among the MCs evaluated for experimental stroke, CBD appears to be the most consistently supported therapeutic choice.

We found that the route of administration was the source of heterogeneity in CBF. Compared with intravenous injection, intraperitoneal administration was a more effective route in improving CBF. Intraperitoneal injection provides a relatively stable and sustained drug absorption process, thereby increasing both the effective concentration and the duration of action (Chaudhary et al., 2010; Al Shoyaib et al., 2019). Moreover, this route was the most frequently employed in the infarct volume subgroup, demonstrating greater robustness of its results. Therefore, intraperitoneal injection may represent a suitable administration method in the MCAO model. Moreover, subgroup analyses of infarct volume and CBF revealed that continuous treatment initiated before and maintained after MCAO achieved the greatest therapeutic efficacy, suggesting that a continuous administration strategy can confer more comprehensive neuroprotection.

### 4.4 Safety, toxicity, and addiction potential

MCs may exert adverse effects in humans. Research indicates that THC-containing medications can cause a range of side effects, including psychiatric and behavioral abnormalities, cognitive impairment, and cardiovascular symptoms (Breijyeh et al., 2021; Velayudhan et al., 2024). Prolonged use results in drug dependence and addiction, with withdrawal symptoms such as irritability, depression, sleep disorders, and decreased appetite emerging after discontinuation (Brown et al., 2021). HU-210 and HU-211 exhibit stronger receptor affinity and agonistic effects, and thus present more significant potential toxicity (Castaneto et al., 2014). We noted that all the included animal studies employed acute or short-term dosing regimens. Therefore, the results of this study cannot provide information on the safety of long-term or repeated administration of MCs in stroke patients. The current evidence fell short of assessing potential chronic toxicity, drug dependence, withdrawal symptoms, and psychiatric and behavioral side effects. Furthermore, the effective doses in animal experiments may not fully correspond to actual human dosages. While the CBD doses used here may correspond to low human levels, the high bioavailability of injectable routes in animals contrasts with the typically lower oral bioavailability in humans (Millar et al., 2019; O'Sullivan et al., 2024). In addition, a dose equivalent to 10 mg/kg THC in rodents is likely to exceed the human psychoactive threshold. A dose of just 10 mg of THC caused acute adverse behavioral and physiological effects in healthy adults (Martin-Santos et al., 2012). Currently, there is a lack of research on safe human doses for synthetic cannabinoids such as HU-210 and HU-211. Therefore, the use of MCs in stroke treatment still poses certain safety issues, and preclinical study results do not imply the absence of clinical risks.

### 4.5 Future prospects and trend analysis

Current research on MCs for ischemic stroke remains largely preclinical. There is a lack of clinical evidence directly illustrating the efficacy and adverse effects of MCs in stroke treatment, and clinical translation faces several challenges. Notably, some preliminary clinical studies have explored the use of MCs in other neurological disorders and stroke-related complications. For instance, MCs, especially CBD, have shown promise in reducing seizure rates among children diagnosed with Dravet syndrome (Treves et al., 2021). A randomized controlled trial for post-stroke spasticity found that nabiximols was safe but did not significantly improve patients’ spasticity symptoms (Marinelli et al., 2022). Additionally, a case study documented that nabiximols could effectively reduce post-stroke pain and significantly improve mood, sleep, and quality of life (Moser, 2021). These clinical findings indicate that certain cannabinoids possess therapeutic potential in specific diseases. However, their results cannot directly confirm the neuroprotective efficacy of MCs in acute stroke, and concerns regarding psychoactive effects, addiction, and long-term toxicity persist. Therefore, before MCs are used for ischemic stroke treatment, rigorously designed clinical trials are essential to systematically evaluate their safety, efficacy, and optimal treatment protocols. Future research should focus on validating the critical steps in translating MCs from bench to bedside, thereby objectively assessing their true potential.

### 4.6 Advantages and limitations

This study provides a comprehensive overview of MCs research in cerebral ischemia by bibliometric analysis and elucidates the collaborative landscape and research trends. Concurrently, the meta-analysis offers robust preclinical evidence supporting the efficacy of MCs in ischemic stroke. However, our study still has certain limitations. Therefore, the results of this study need to be interpreted with caution.

In the bibliometric section, the analysis software constrained our search to the WoSCC, PubMed, and Scopus databases, possibly omitting relevant literature from other sources. Since all included articles were published in English and those not meeting the criteria were manually excluded, this process may have resulted in selection bias. Given the inclusion of multiple cerebral ischemia models in the study, caution is required when generalizing the findings to a single model. Moreover, there are challenges in illuminating the mechanistic differences between these models.

The limitations of the meta-analysis section are as follows. First, the methodological quality of the included studies was generally low. Most studies were found to have flaws in the risk of bias assessment, particularly the lack of detailed description in randomization, allocation concealment, and blinding implementation. In addition, none of the included studies conducted safety evaluations.

Second, potential biases may be introduced during data extraction and analysis. The extraction of raw data from some figures using WebPlotDigitizer software may have led to measurement bias. Meanwhile, significant heterogeneity was observed across the included studies. However, subgroup analyses were insufficient to fully reveal its potential sources. This heterogeneity likely stems from various biological and methodological confounders, such as differences in animal species, modeling techniques, surgical standardization, and the underrepresentation of female animals. Furthermore, overall study quality limitations and the presence of publication bias may have compounded methodological variability. Future research should therefore adopt more rigorous and standardized designs to clarify the impact of these confounders on treatment outcomes.

Furthermore, more studies on CBD were included. Our results indicated that CBD exhibited the most promising therapeutic potential among all compounds evaluated. However, this conclusion was drawn based on the imbalance in the number of studies between different cannabinoids, which could introduce potential bias when interpreting comparative results. Further research should be conducted to elucidate the effects of other MCs, thereby enhancing the quality and credibility of the findings.

Finally, standardized rodent models cannot fully simulate the complex pathophysiological processes of human stroke. Most included studies used healthy animals to establish focal ischemia models, lacking common comorbidities like diabetes, atherosclerosis, hypertension, and hyperlipidemia.

## 5 Conclusion

The bibliometric findings reveal a rapidly evolving field with growing global contributions, though greater international collaboration is encouraged. Current research hotspots focus on neuroprotective mechanisms, pathological models, and the screening of bioactive components. Moreover, the results of meta-analysis consolidate preclinical evidence, demonstrating that MCs confer neuroprotection by mitigating multiple pathological processes, including cerebral tissue perfusion, BBB permeability and cerebral edema, oxidative stress, excitotoxicity, inflammatory responses, and apoptosis. However, translating this promise into clinical reality necessitates rigorously designed clinical trials. Future efforts should focus on bridging this translational gap to fully validate the efficacy and safety of MCs, thereby offering critical insights for the development of novel therapies for ischemic stroke.

## Data Availability

The original contributions presented in the study are included in the article/Supplementary material, further inquiries can be directed to the corresponding author.

## References

[ref1] Afzal S. Abdul Manap A. S. Attiq A. Albokhadaim I. Kandeel M. Alhojaily S. M. (2023). From imbalance to impairment: the central role of reactive oxygen species in oxidative stress-induced disorders and therapeutic exploration. Front. Pharmacol. 14:1269581. doi: 10.3389/fphar.2023.1269581, 37927596 PMC10622810

[ref2] Al Shoyaib A. Archie S. R. Karamyan V. T. (2019). Intraperitoneal route of drug administration: should it be used in experimental animal studies? Pharm. Res. 37:12. doi: 10.1007/s11095-019-2745-x, 31873819 PMC7412579

[ref3] Alsbrook D. L. Di Napoli M. Bhatia K. Biller J. Andalib S. Hinduja A. . (2023). Neuroinflammation in acute ischemic and hemorrhagic stroke. Curr. Neurol. Neurosci. Rep. 23, 407–431. doi: 10.1007/s11910-023-01282-2, 37395873 PMC10544736

[ref4] Amin M. R. Ali D. W. (2019). Pharmacology of medical Cannabis. Adv. Exp. Med. Biol. 1162, 151–165. doi: 10.1007/978-3-030-21737-2_8, 31332738

[ref5] Barthels D. Das H. (2020). Current advances in ischemic stroke research and therapies. Biochim. Biophys. Acta Mol. basis Dis. 1866:165260. doi: 10.1016/j.bbadis.2018.09.012, 31699365 PMC6981280

[ref6] Benyó Z. Ruisanchez É. Leszl-Ishiguro M. Sándor P. Pacher P. (2016). Endocannabinoids in cerebrovascular regulation. Am. J. Physiol. Heart Circ. Physiol. 310, H785–H801. doi: 10.1152/ajpheart.00571.2015, 26825517 PMC4865067

[ref7] Breijyeh Z. Jubeh B. Bufo S. A. Karaman R. Scrano L. (2021). Cannabis: A toxin-producing plant with potential therapeutic uses. Toxins 13. doi: 10.3390/toxins13020117, 33562446 PMC7915118

[ref8] Brown J. D. Rivera Rivera K. J. Hernandez L. Y. C. Doenges M. R. Auchey I. Pham T. . (2021). Natural and synthetic cannabinoids: pharmacology, uses, adverse drug events, and drug interactions. J. Clin. Pharmacol. 61, S37–s52. doi: 10.1002/jcph.1871, 34396558

[ref9] Cai M. Yang Q. Li G. Sun S. Chen Y. Tian L. . (2017). Activation of cannabinoid receptor 1 is involved in protection against mitochondrial dysfunction and cerebral ischaemic tolerance induced by isoflurane preconditioning. Br. J. Anaesth. 119, 1213–1223. doi: 10.1093/bja/aex267, 29045576

[ref10] Candelario-Jalil E. Dijkhuizen R. M. Magnus T. (2022). Neuroinflammation, stroke, blood-brain barrier dysfunction, and imaging modalities. Stroke 53, 1473–1486. doi: 10.1161/strokeaha.122.036946, 35387495 PMC9038693

[ref11] Castaneto M. S. Gorelick D. A. Desrosiers N. A. Hartman R. L. Pirard S. Huestis M. A. (2014). Synthetic cannabinoids: epidemiology, pharmacodynamics, and clinical implications. Drug Alcohol Depend. 144, 12–41. doi: 10.1016/j.drugalcdep.2014.08.005, 25220897 PMC4253059

[ref12] Castillo-Arellano J. Canseco-Alba A. Cutler S. J. León F. (2023). The Polypharmacological effects of Cannabidiol. Molecules 28. doi: 10.3390/molecules28073271, 37050032 PMC10096752

[ref13] Ceprián M. Jiménez-Sánchez L. Vargas C. Barata L. Hind W. Martínez-Orgado J. (2017). Cannabidiol reduces brain damage and improves functional recovery in a neonatal rat model of arterial ischemic stroke. Neuropharmacology 116, 151–159. doi: 10.1016/j.neuropharm.2016.12.017, 28012949

[ref14] Chaudhary K. Haddadin S. Nistala R. Papageorgio C. (2010). Intraperitoneal drug therapy: an advantage. Curr. Clin. Pharmacol. 5, 82–88. doi: 10.2174/157488410791110779, 20156151

[ref15] Chen C. M. (2006). CiteSpace II: detecting and visualizing emerging trends and transient patterns in scientific literature. J. Am. Soc. Inf. Sci. Technol. 57, 359–377. doi: 10.1002/asi.20317

[ref16] Chen K. Xu B. Xiao X. Long L. Zhao Q. Fang Z. . (2024). Involvement of CKS1B in the anti-inflammatory effects of cannabidiol in experimental stroke models. Exp. Neurol. 373:114654. doi: 10.1016/j.expneurol.2023.114654, 38104887

[ref17] Chen H. S. Zhao Z. A. Shen X. Y. Qiu S. Q. Cui Y. Qiu J. . (2025). Edaravone dexborneol for ischemic stroke with sufficient recanalization after thrombectomy: a randomized phase II trial. Nat. Commun. 16:2393. doi: 10.1038/s41467-025-57774-x, 40064868 PMC11894225

[ref18] de Souza Stork S. Mathias K. Gava F. Joaquim L. Dos Santos D. Tiscoski A. D. B. . (2025). Full-spectrum *Cannabis sativa* extract enhances gut-peripheral organ integrity after experimental ischemic stroke. Inflammopharmacology 33, 3279–3305. doi: 10.1007/s10787-025-01775-1, 40389682

[ref19] DeLong J. H. Ohashi S. N. O'Connor K. C. Sansing L. H. (2022). Inflammatory responses after ischemic stroke. Semin. Immunopathol. 44, 625–648. doi: 10.1007/s00281-022-00943-7, 35767089

[ref20] Deng X. Zhao M. Zhang E. Wei L. Gao X. Zhang D. . (2025). New insights into acute ischemic stroke from the perspective of spatial omics. Theranostics 15, 7902–7924. doi: 10.7150/thno.113396, 40756344 PMC12316105

[ref21] Durmaz R. Ozden H. Kanbak G. Aral E. Arslan O. C. Kartkaya K. . (2008). The protective effect of dexanabinol (HU-211) on nitric oxide and cysteine protease-mediated neuronal death in focal cerebral ischemia. Neurochem. Res. 33, 1683–1691. doi: 10.1007/s11064-008-9605-0, 18404379

[ref22] Endres M. Moro M. A. Nolte C. H. Dames C. Buckwalter M. S. Meisel A. (2022). Immune pathways in etiology, acute phase, and chronic sequelae of ischemic stroke. Circ. Res. 130, 1167–1186. doi: 10.1161/circresaha.121.319994, 35420915

[ref23] Feigin V. L. Brainin M. Norrving B. Martins S. O. Pandian J. Lindsay P. . (2025). World stroke organization: global stroke fact sheet 2025. Int. J. Stroke 20, 132–144. doi: 10.1177/17474930241308142, 39635884 PMC11786524

[ref24] Feigin V. L. Nguyen G. Cercy K. Johnson C. O. Alam T. Parmar P. G. . (2018). Global, regional, and country-specific lifetime risks of stroke, 1990 and 2016. N. Engl. J. Med. 379, 2429–2437. doi: 10.1056/NEJMoa1804492, 30575491 PMC6247346

[ref25] Fernández-Ruiz J. Moro M. A. Martínez-Orgado J. (2015). Cannabinoids in neurodegenerative disorders and stroke/brain trauma: from preclinical models to clinical applications. Neurotherapeutics 12, 793–806. doi: 10.1007/s13311-015-0381-7, 26260390 PMC4604192

[ref26] Fluri F. Schuhmann M. K. Kleinschnitz C. (2015). Animal models of ischemic stroke and their application in clinical research. Drug Des. Devel. Ther. 9, 3445–3454. doi: 10.2147/dddt.S56071, 26170628 PMC4494187

[ref27] GBD 2021 Stroke Risk Factor Collaborators (2024). Global, regional, and national burden of stroke and its risk factors, 1990-2021: a systematic analysis for the global burden of disease study 2021. Lancet Neurol. 23, 973–1003. doi: 10.1016/s1474-4422(24)00369-7, 39304265 PMC12254192

[ref28] Gilbert G. L. Kim H. J. Waataja J. J. Thayer S. A. (2007). Delta9-tetrahydrocannabinol protects hippocampal neurons from excitotoxicity. Brain Res. 1128, 61–69. doi: 10.1016/j.brainres.2006.03.011, 17140550

[ref29] Hampson A. J. Grimaldi M. Axelrod J. Wink D. (1998). Cannabidiol and (−)Delta9-tetrahydrocannabinol are neuroprotective antioxidants. Proc. Natl. Acad. Sci. USA 95, 8268–8273. doi: 10.1073/pnas.95.14.8268, 9653176 PMC20965

[ref30] Hayakawa K. Irie K. Sano K. Watanabe T. Higuchi S. Enoki M. . (2009). Therapeutic time window of cannabidiol treatment on delayed ischemic damage via high-mobility group box1-inhibiting mechanism. Biol. Pharm. Bull. 32, 1538–1544. doi: 10.1248/bpb.32.1538, 19721229

[ref31] Hayakawa K. Mishima K. Abe K. Hasebe N. Takamatsu F. Yasuda H. . (2004). Cannabidiol prevents infarction via the non-CB1 cannabinoid receptor mechanism. Neuroreport 15, 2381–2385. doi: 10.1097/00001756-200410250-00016, 15640760

[ref32] Hayakawa K. Mishima K. Irie K. Hazekawa M. Mishima S. Fujioka M. . (2008). Cannabidiol prevents a post-ischemic injury progressively induced by cerebral ischemia via a high-mobility group box1-inhibiting mechanism. Neuropharmacology 55, 1280–1286. doi: 10.1016/j.neuropharm.2008.06.040, 18634812

[ref33] Hayakawa K. Mishima K. Nozako M. Hazekawa M. Irie K. Fujioka M. . (2007a). Delayed treatment with cannabidiol has a cerebroprotective action via a cannabinoid receptor-independent myeloperoxidase-inhibiting mechanism. J. Neurochem. 102, 1488–1496. doi: 10.1111/j.1471-4159.2007.04565.x 17437545

[ref34] Hayakawa K. Mishima K. Nozako M. Hazekawa M. Ogata A. Fujioka M. . (2007b). Delta9-tetrahydrocannabinol (Delta9-THC) prevents cerebral infarction via hypothalamic-independent hypothermia. Life Sci. 80, 1466–1471. doi: 10.1016/j.lfs.2007.01.014 17289082

[ref35] Hayakawa K. Mishima K. Nozako M. Ogata A. Hazekawa M. Liu A. X. . (2007c). Repeated treatment with cannabidiol but not Δ9-tetrahydrocannabinol has a neuroprotective effect without the development of tolerance. Neuropharmacology 52, 1079–1087. doi: 10.1016/j.neuropharm.2006.11.005, 17320118

[ref36] Henshaw F. R. Dewsbury L. S. Lim C. K. Steiner G. Z. (2021). The effects of cannabinoids on pro- and anti-inflammatory cytokines: A systematic review of in vivo studies. Cannabis Cannabinoid Res. 6, 177–195. doi: 10.1089/can.2020.0105, 33998900 PMC8266561

[ref37] Hidding U. Mainka T. Buhmann C. (2024). Therapeutic use of medical Cannabis in neurological diseases: a clinical update. J. Neural Transm. (Vienna) 131, 117–126. doi: 10.1007/s00702-023-02719-1, 38015317 PMC10791790

[ref38] Hillman T. C. Matei N. Tang J. Zhang J. H. (2019). Developing a standardized system of exposure and intervention endpoints for isoflurane in preclinical stroke models. Med. Gas Res. 9, 46–51. doi: 10.4103/2045-9912.254640, 30950418 PMC6463442

[ref39] Hind W. H. England T. J. O'Sullivan S. E. (2016). Cannabidiol protects an in vitro model of the blood-brain barrier from oxygen-glucose deprivation via PPARγ and 5-HT1A receptors. Br. J. Pharmacol. 173, 815–825. doi: 10.1111/bph.13368, 26497782 PMC4761095

[ref40] Hoch E. Volkow N. D. Friemel C. M. Lorenzetti V. Freeman T. P. Hall W. (2025). Cannabis, cannabinoids and health: a review of evidence on risks and medical benefits. Eur. Arch. Psychiatry Clin. Neurosci. 275, 281–292. doi: 10.1007/s00406-024-01880-2, 39299947 PMC11910417

[ref41] Hooijmans C. R. Rovers M. M. de Vries R. B. Leenaars M. Ritskes-Hoitinga M. Langendam M. W. (2014). SYRCLE'S risk of bias tool for animal studies. BMC Med. Res. Methodol. 14:43. doi: 10.1186/1471-2288-14-43, 24667063 PMC4230647

[ref42] Janssen B. J. De Celle T. Debets J. J. Brouns A. E. Callahan M. F. Smith T. L. (2004). Effects of anesthetics on systemic hemodynamics in mice. Am. J. Physiol. Heart Circ. Physiol. 287, H1618–H1624. doi: 10.1152/ajpheart.01192.2003, 15155266

[ref43] Ji Y. Gao Q. Ma Y. Wang F. Tan X. Song D. . (2023). An MMP-9 exclusive neutralizing antibody attenuates blood-brain barrier breakdown in mice with stroke and reduces stroke patient-derived MMP-9 activity. Pharmacol. Res. 190:106720. doi: 10.1016/j.phrs.2023.106720, 36893823 PMC11934118

[ref44] Khaksar S. Bigdeli M. R. (2017a). Anti-excitotoxic effects of cannabidiol are partly mediated by enhancement of NCX2 and NCX3 expression in animal model of cerebral ischemia. Eur. J. Pharmacol. 794, 270–279. doi: 10.1016/j.ejphar.2016.11.011, 27856160

[ref45] Khaksar S. Bigdeli M. R. (2017b). Intra-cerebral cannabidiol infusion-induced neuroprotection is partly associated with the TNF-α/TNFR1/NF-кB pathway in transient focal cerebral ischaemia. Brain Inj. 31, 1932–1943. doi: 10.1080/02699052.2017.1358397, 28872345

[ref46] Khaksar S. Bigdeli M. R. (2017c). Correlation between Cannabidiol-induced reduction of infarct volume and inflammatory factors expression in ischemic stroke model. Basic Clin Neurosci 8, 139–146. doi: 10.18869/nirp.bcn.8.2.139, 28539998 PMC5440923

[ref47] Khaksar S. Bigdeli M. Samiee A. Shirazi-Zand Z. (2022). Antioxidant and anti-apoptotic effects of cannabidiol in model of ischemic stroke in rats. Brain Res. Bull. 180, 118–130. doi: 10.1016/j.brainresbull.2022.01.001, 35031355

[ref48] Kitano H. Kirsch J. R. Hurn P. D. Murphy S. J. (2007). Inhalational anesthetics as neuroprotectants or chemical preconditioning agents in ischemic brain. J. Cereb. Blood Flow Metab. 27, 1108–1128. doi: 10.1038/sj.jcbfm.9600410, 17047683 PMC2266688

[ref49] Kumari S. Dhapola R. Sharma P. Nagar P. Medhi B. HariKrishnaReddy D. (2024). The impact of cytokines in neuroinflammation-mediated stroke. Cytokine Growth Factor Rev. 78, 105–119. doi: 10.1016/j.cytogfr.2024.06.002, 39004599

[ref50] Lavayen B. P. Yang C. Larochelle J. Liu L. Tishko R. J. de Oliveira A. C. P. . (2023). Neuroprotection by the cannabidiol aminoquinone VCE-004.8 in experimental ischemic stroke in mice. Neurochem. Int. 165:105508. doi: 10.1016/j.neuint.2023.105508, 36863495

[ref51] Lavie G. Teichner A. Shohami E. Ovadia H. Leker R. R. (2001). Long term cerebroprotective effects of dexanabinol in a model of focal cerebral ischemia. Brain Res. 901, 195–201. doi: 10.1016/s0006-8993(01)02356-3, 11368967

[ref52] Leker R. R. Gai N. Mechoulam R. Ovadia H. (2003). Drug-induced hypothermia reduces ischemic damage: effects of the cannabinoid HU-210. Stroke 34, 2000–2006. doi: 10.1161/01.Str.0000079817.68944.1e, 12829867

[ref53] Li Z. Bi R. Sun S. Chen S. Chen J. Hu B. . (2022). The role of oxidative stress in acute ischemic stroke-related thrombosis. Oxidative Med. Cell. Longev. 2022:8418820. doi: 10.1155/2022/8418820, 36439687 PMC9683973

[ref54] Li C. Luo Y. Li S. (2026). Mechanistic insights of neuronal death and neuroprotective therapeutic approaches in stroke. Neural Regen. Res. 21, 869–886. doi: 10.4103/nrr.Nrr-d-24-01324, 40313116 PMC12296499

[ref55] Liu S. Xu J. Liu Y. You Y. Xie L. Tong S. . (2022). Neutrophil-biomimetic "Nanobuffer" for remodeling the microenvironment in the infarct Core and protecting neurons in the penumbra via neutralization of detrimental factors to treat ischemic stroke. ACS Appl. Mater. Interfaces 14, 27743–27761. doi: 10.1021/acsami.2c09020, 35695238

[ref56] Maayah Z. H. Takahara S. Ferdaoussi M. Dyck J. R. B. (2020). The molecular mechanisms that underpin the biological benefits of full-spectrum cannabis extract in the treatment of neuropathic pain and inflammation. Biochim. Biophys. Acta Mol. basis Dis. 1866:165771. doi: 10.1016/j.bbadis.2020.165771, 32201189

[ref57] Marinelli L. Puce L. Mori L. Leandri M. Rosa G. M. Currà A. . (2022). Cannabinoid effect and safety in spasticity following stroke: A double-blind randomized placebo-controlled study. Front. Neurol. 13:892165. doi: 10.3389/fneur.2022.892165, 35812088 PMC9261779

[ref58] Martinez Naya N. Kelly J. Corna G. Golino M. Abbate A. Toldo S. (2023). Molecular and cellular mechanisms of action of Cannabidiol. Molecules 28. doi: 10.3390/molecules28165980, 37630232 PMC10458707

[ref59] Martin-Santos R. Crippa J. A. Batalla A. Bhattacharyya S. Atakan Z. Borgwardt S. . (2012). Acute effects of a single, oral dose of d9-tetrahydrocannabinol (THC) and cannabidiol (CBD) administration in healthy volunteers. Curr. Pharm. Des. 18, 4966–4979. doi: 10.2174/138161212802884780, 22716148

[ref60] Maud P. Thavarak O. Cédrick L. Michèle B. Vincent B. Olivier P. . (2014). Evidence for the use of isoflurane as a replacement for chloral hydrate anesthesia in experimental stroke: an ethical issue. Biomed. Res. Int. 2014:802539. doi: 10.1155/2014/802539, 24719888 PMC3955691

[ref61] Meyer E. Rieder P. Gobbo D. Candido G. Scheller A. de Oliveira R. M. W. . (2022). Cannabidiol exerts a neuroprotective and glia-balancing effect in the subacute phase of stroke. Int. J. Mol. Sci. 23. doi: 10.3390/ijms232112886, 36361675 PMC9659180

[ref62] Millar S. A. Stone N. L. Bellman Z. D. Yates A. S. England T. J. O'Sullivan S. E. (2019). A systematic review of cannabidiol dosing in clinical populations. Br. J. Clin. Pharmacol. 85, 1888–1900. doi: 10.1111/bcp.14038, 31222854 PMC6710502

[ref63] Mishima K. Hayakawa K. Abe K. Ikeda T. Egashira N. Iwasaki K. . (2005). Cannabidiol prevents cerebral infarction via a serotonergic 5-hydroxytryptamine1A receptor-dependent mechanism. Stroke 36, 1077–1082. doi: 10.1161/01.Str.0000163083.59201.34 15845890

[ref64] Moher D. Liberati A. Tetzlaff J. Altman D. G. (2009). Preferred reporting items for systematic reviews and meta-analyses: the PRISMA statement. BMJ 339:b2535. doi: 10.1136/bmj.b2535, 19622551 PMC2714657

[ref65] Moser U. (2021). Tetrahydrocannabinol and cannabidiol as an oromucosal spray in a 1:1 ratio: a therapeutic option for patients with central post-stroke pain syndrome? BMJ Case Rep. 14. doi: 10.1136/bcr-2021-243072, 34230048 PMC8264571

[ref66] Mussbacher M. Derler M. Basílio J. Schmid J. A. (2023). NF-κB in monocytes and macrophages - an inflammatory master regulator in multitalented immune cells. Front. Immunol. 14:1134661. doi: 10.3389/fimmu.2023.1134661, 36911661 PMC9995663

[ref67] Navarrete C. García-Martín A. Correa-Sáez A. Prados M. E. Fernández F. Pineda R. . (2022). A cannabidiol aminoquinone derivative activates the PP2A/B55α/HIF pathway and shows protective effects in a murine model of traumatic brain injury. J. Neuroinflammation 19:177. doi: 10.1186/s12974-022-02540-9, 35810304 PMC9270745

[ref68] Ogunbiyi M. O. Hindocha C. Freeman T. P. Bloomfield M. A. P. (2020). Acute and chronic effects of Δ(9)-tetrahydrocannabinol (THC) on cerebral blood flow: a systematic review. Prog. Neuro-Psychopharmacol. Biol. Psychiatry 101:109900. doi: 10.1016/j.pnpbp.2020.109900, 32109508

[ref69] Oh S. S. Narver H. L. (2024). Mouse and rat anesthesia and analgesia. Curr. Protoc. 4:e995. doi: 10.1002/cpz1.995, 38406895 PMC10914332

[ref70] O'Sullivan S. E. Jensen S. S. Kolli A. R. Nikolajsen G. N. Bruun H. Z. Hoeng J. (2024). Strategies to improve cannabidiol bioavailability and drug delivery. Pharmaceuticals (Basel) 17. doi: 10.3390/ph17020244 PMC10892205 38399459

[ref71] Pawluk H. Tafelska-Kaczmarek A. Sopońska M. Porzych M. Modrzejewska M. Pawluk M. . (2024). The influence of oxidative stress markers in patients with ischemic stroke. Biomolecules 14. doi: 10.3390/biom14091130, 39334896 PMC11430825

[ref72] Qiu Y. M. Zhang C. L. Chen A. Q. Wang H. L. Zhou Y. F. Li Y. N. . (2021). Immune cells in the BBB disruption after acute ischemic stroke: targets for immune therapy? Front. Immunol. 12:678744. doi: 10.3389/fimmu.2021.678744, 34248961 PMC8260997

[ref73] Raïch I. Lillo J. Rivas-Santisteban R. Rebassa J. B. Capó T. Santandreu M. . (2024). Potential of CBD acting on cannabinoid receptors CB(1) and CB(2) in ischemic stroke. Int. J. Mol. Sci. 25. doi: 10.3390/ijms25126708, 38928415 PMC11204117

[ref74] Richter J. S. Quenardelle V. Rouyer O. Raul J. S. Beaujeux R. Gény B. . (2018). A systematic review of the complex effects of cannabinoids on cerebral and peripheral circulation in animal models. Front. Physiol. 9:622. doi: 10.3389/fphys.2018.00622, 29896112 PMC5986896

[ref75] Rodríguez-Muñoz M. Onetti Y. Cortés-Montero E. Garzón J. Sánchez-Blázquez P. (2018). Cannabidiol enhances morphine antinociception, diminishes NMDA-mediated seizures and reduces stroke damage via the sigma 1 receptor. Mol. Brain 11:51. doi: 10.1186/s13041-018-0395-2, 30223868 PMC6142691

[ref76] San Luis C. V. O'Hana S. N. C. Shekhar S. Sugg R. Villareal D. J. Mehta T. . (2020). Association between recent cannabinoid use and acute ischemic stroke. Neurol. Clin. Pract. 10, 333–339. doi: 10.1212/cpj.0000000000000888 32983613 PMC7508332

[ref77] Sarraf-Yazdi S. Sheng H. Miura Y. McFarlane C. Dexter F. Pearlstein R. . (1998). Relative neuroprotective effects of dizocilpine and isoflurane during focal cerebral ischemia in the rat. Anesth. Analg. 87, 72–78. doi: 10.1097/00000539-199807000-00016, 9661549

[ref78] Scharf E. L. (2017). Translating endocannabinoid biology into clinical practice: Cannabidiol for stroke prevention. Cannabis Cannabinoid Res. 2, 259–264. doi: 10.1089/can.2017.0033, 29098188 PMC5665427

[ref79] Scheid S. Goebel U. Ulbrich F. (2023). Neuroprotection is in the air-inhaled gases on their way to the neurons. Cells 12. doi: 10.3390/cells12202480, 37887324 PMC10605176

[ref80] Shi Y. H. Li Y. Wang Y. Xu Z. Fu H. Zheng G. Q. (2020). Ginsenoside-Rb1 for ischemic stroke: A systematic review and Meta-analysis of preclinical evidence and possible mechanisms. Front. Pharmacol. 11:285. doi: 10.3389/fphar.2020.00285, 32296332 PMC7137731

[ref81] Singh K. Bhushan B. Chanchal D. K. Sharma S. K. Rani K. Yadav M. K. . (2023). Emerging therapeutic potential of Cannabidiol (CBD) in neurological disorders: A comprehensive review. Behav. Neurol. 2023, 1–17. doi: 10.1155/2023/8825358, 37868743 PMC10586905

[ref82] Sommer C. J. (2017). Ischemic stroke: experimental models and reality. Acta Neuropathol. 133, 245–261. doi: 10.1007/s00401-017-1667-0, 28064357 PMC5250659

[ref83] Stone N. L. Murphy A. J. England T. J. O'Sullivan S. E. (2020). A systematic review of minor phytocannabinoids with promising neuroprotective potential. Br. J. Pharmacol. 177, 4330–4352. doi: 10.1111/bph.15185, 32608035 PMC7484504

[ref84] Suliman N. A. Taib C. N. M. Moklas M. A. M. Basir R. (2018). Delta-9-tetrahydrocannabinol (∆(9)-THC) induce neurogenesis and improve cognitive performances of male Sprague Dawley rats. Neurotox. Res. 33, 402–411. doi: 10.1007/s12640-017-9806-x, 28933048 PMC5766723

[ref85] Sultan S. R. Millar S. A. England T. J. O'Sullivan S. E. (2017). A systematic review and Meta-analysis of the Haemodynamic effects of Cannabidiol. Front. Pharmacol. 8:81. doi: 10.3389/fphar.2017.00081, 28286481 PMC5323388

[ref86] Sultan S. R. Millar S. A. O'Sullivan S. E. England T. J. (2018). A systematic review and meta-analysis of the in vivo haemodynamic effects of Δ^8^-tetrahydrocannabinol. Pharmaceuticals (Basel) 11. doi: 10.3390/ph11010013 PMC5874709 29385080

[ref87] Sun S. Hu F. Wu J. Zhang S. (2017). Cannabidiol attenuates OGD/R-induced damage by enhancing mitochondrial bioenergetics and modulating glucose metabolism via pentose-phosphate pathway in hippocampal neurons. Redox Biol. 11, 577–585. doi: 10.1016/j.redox.2016.12.029, 28110213 PMC5247568

[ref88] Teichner A. Ovadia H. Lavie G. Leker R. R. (2003). Combination of dexanabinol and tempol in focal cerebral ischemia: is there a ceiling effect? Exp. Neurol. 182, 353–360. doi: 10.1016/s0014-4886(03)00083-9, 12895446

[ref89] Tirandi A. Sgura C. Carbone F. Montecucco F. Liberale L. (2023). Inflammatory biomarkers of ischemic stroke. Intern. Emerg. Med. 18, 723–732. doi: 10.1007/s11739-023-03201-2, 36745280 PMC10082112

[ref90] Treves N. Mor N. Allegaert K. Bassalov H. Berkovitch M. Stolar O. E. . (2021). Efficacy and safety of medical cannabinoids in children: a systematic review and meta-analysis. Sci. Rep. 11:23462. doi: 10.1038/s41598-021-02770-6, 34873203 PMC8648720

[ref91] Uzdensky A. B. (2019). Apoptosis regulation in the penumbra after ischemic stroke: expression of pro- and antiapoptotic proteins. Apoptosis 24, 687–702. doi: 10.1007/s10495-019-01556-6, 31256300

[ref92] van Eck N. J. Waltman L. (2010). Software survey: VOSviewer, a computer program for bibliometric mapping. Scientometrics 84, 523–538. doi: 10.1007/s11192-009-0146-3, 20585380 PMC2883932

[ref93] Velayudhan L. Pisani S. Dugonjic M. McGoohan K. Bhattacharyya S. (2024). Adverse events caused by cannabinoids in middle aged and older adults for all indications: a meta-analysis of incidence rate difference. Age Ageing 53. doi: 10.1093/ageing/afae261, 39602500 PMC11601816

[ref94] Vicente-Acosta A. Ceprian M. Sobrino P. Pazos M. R. Loría F. (2022). Cannabinoids as glial cell modulators in ischemic stroke: implications for neuroprotection. Front. Pharmacol. 13:888222. doi: 10.3389/fphar.2022.888222, 35721207 PMC9199389

[ref95] Villa M. Martínez-Vega M. Silva L. Muneta-Arrate I. Gómez-Soria A. Muguruza C. . (2024a). Effects of cannabidiol in post-stroke mood disorders in neonatal rats. Pediatr. Res. 95, 1783–1790. doi: 10.1038/s41390-024-03077-8, 38360979

[ref96] Villa M. Martínez-Vega M. Silva L. Romero A. de Hoz-Rivera M. Prados M. E. . (2024b). Neuroprotective effects of VCE-004.8 in a rat model of neonatal stroke. Eur. J. Pharmacol. 972:176554. doi: 10.1016/j.ejphar.2024.176554 38582276

[ref97] Vitale I. Pietrocola F. Guilbaud E. Aaronson S. A. Abrams J. M. Adam D. . (2023). Apoptotic cell death in disease-current understanding of the NCCD 2023. Cell Death Differ. 30, 1097–1154. doi: 10.1038/s41418-023-01153-w, 37100955 PMC10130819

[ref98] Wang A. Jia B. Zhang X. Huo X. Chen J. Gui L. . (2023). Efficacy and safety of butylphthalide in patients with acute ischemic stroke: A randomized clinical trial. JAMA Neurol. 80, 851–859. doi: 10.1001/jamaneurol.2023.1871, 37358859 PMC10294018

[ref99] Wang Y. Li M. Jiang Y. Ji Q. (2024). Comparative efficacy of neuroprotective agents for improving neurological function and prognosis in acute ischemic stroke: a network meta-analysis. Front. Neurosci. 18:1530987. doi: 10.3389/fnins.2024.1530987, 39834702 PMC11743486

[ref100] Whiting P. F. Wolff R. F. Deshpande S. Di Nisio M. Duffy S. Hernandez A. V. . (2015). Cannabinoids for medical use: a systematic review and meta-analysis. JAMA 313, 2456–2473. doi: 10.1001/jama.2015.6358, 26103030

[ref101] Wu J. Jia J. Ji D. Jiao W. Huang Z. Zhang Y. (2023). Advances in nitric oxide regulators for the treatment of ischemic stroke. Eur. J. Med. Chem. 262:115912. doi: 10.1016/j.ejmech.2023.115912, 37931330

[ref102] Xu B. T. Li M. F. Chen K. C. Li X. Cai N. B. Xu J. P. . (2023). Mitofusin-2 mediates cannabidiol-induced neuroprotection against cerebral ischemia in rats. Acta Pharmacol. Sin. 44, 499–512. doi: 10.1038/s41401-022-01004-3, 36229600 PMC9958179

[ref103] Xu L. Mi Y. Meng Q. Liu Y. Wang F. Zhang G. . (2024). Anti-inflammatory effects of quinolinyl analog of resveratrol targeting TLR4 in MCAO/R ischemic stroke rat model. Phytomedicine 128:155344. doi: 10.1016/j.phymed.2024.155344, 38493721

[ref104] Yagami T. Yamamoto Y. Koma H. (2019). Pathophysiological roles of intracellular proteases in neuronal development and neurological diseases. Mol. Neurobiol. 56, 3090–3112. doi: 10.1007/s12035-018-1277-4, 30097848

[ref105] Yang X. M. Yu H. Li J. X. Li N. Li C. Xu D. H. . (2024). Excitotoxic storms of ischemic stroke: A non-neuronal perspective. Mol. Neurobiol. 61, 9562–9581. doi: 10.1007/s12035-024-04184-7, 38662299

[ref106] Yokubaitis C. G. Jessani H. N. Li H. Amodea A. K. Ward S. J. (2021). Effects of Cannabidiol and Beta-Caryophyllene alone or in combination in a mouse model of permanent ischemia. Int. J. Mol. Sci. 22. doi: 10.3390/ijms22062866, 33799861 PMC7999270

[ref107] Zhu J. Mo J. Liu K. Chen Q. Li Z. He Y. . (2024). Glymphatic system impairment contributes to the formation of brain edema after ischemic stroke. Stroke 55, 1393–1404. doi: 10.1161/strokeaha.123.045941, 38533660

