# Supplementary material for: Exploring the neuroprotective effects and underlying mechanisms of medical cannabinoids in ischemic stroke: a systematic meta-analysis with bibliometric mapping of cerebral ischemia research
Source: Front Neurosci. 2026 Jan 2;19:1731738. doi: 10.3389/fnins.2025.1731738 (PMC12808455; doi:10.3389/fnins.2025.1731738)
Supplement: Supplementary file 1 [file Data_Sheet_1.docx]

**Supplementary Table 1:** Search strategies.

| **Database** | **Search strategy** | **Results** |
| --- | --- | --- |
| Web of science | (TS=("stroke" OR "apoplexy" OR "ischemic stroke*" OR "ischaemic stroke*" OR "brain ischemia" OR "cerebral ischemia" OR "cerebral infarct" OR "brain infarct" OR "intracranial embolism" OR "cerebrovascular accident" OR "brain vascular accident" OR "cerebral infarction" OR "brain ischaemia" OR "brain infarction" OR "ischemic encephalopathy" OR "stroke, ischemic" OR "stroke, ischaemic" OR "acute IS" OR "acute ISs" OR "cerebral stem ischemia" OR "acute ischemic stroke" OR "cryptogenic ischemic stroke" OR "cryptogenic stroke" OR "cryptogenic embolism stroke" OR "wake up stroke" OR "cerebral ischemia reperfusion" OR "reperfusion injury" OR "reperfusion damage" OR "cerebral embolism" OR "cerebral emboli" OR "cerebral embolus" OR "brain embolism" OR "brain emboli" OR "brain embolus")) AND TS=("cannabinoid*" OR "cannabidiol" OR "tetrahydrocannabinol" OR "dronabinol" OR "CBD" OR "THC" OR "epidiolex" OR "epidyolex" OR "marinol" OR "syndros" OR "cannabinol" OR "nabilone" OR "cesamet" OR "canemes" OR "dexanabinol" OR "nabiximols" OR "sativex" OR "cannabigerol" OR "cannabichromene" OR "HU 210" OR "HU 211" OR "cannabidivarin" OR "medical marijuana" OR "medical cannabis" OR "medicinal marijuana" OR "medicinal cannabis" OR "medical cannabinoid*" OR "exocannabinoid*" OR "phytocannabinoid*" OR "phyto-cannabinoid*" OR "delta(9) THC" OR "delta(1) tetrahydrocannabinol" OR "delta(1) THC" OR "delta(9) tetrahydrocannabinol") | 931 |
| PubMed | (("stroke"[Mesh] OR "ischemic stroke"[Mesh] OR "brain ischemia"[Mesh] OR "cerebral infarction"[Mesh] OR "brain infarction"[Mesh] OR "intracranial embolism"[Mesh]) AND ((((("cannabinoids"[Mesh]) OR "cannabidiol"[Mesh]) OR "dronabinol"[Mesh]) OR "cannabinol"[Mesh]) OR "medical marijuana"[Mesh])) OR ((("stroke*"[Title/Abstract] OR "cerebrovascular accident*"[Title/Abstract] OR "cerebral stroke*"[Title/Abstract] OR "cerebrovascular apoplexy"[Title/Abstract] OR "apoplexy"[Title/Abstract] OR "brain vascular accident"[Title/Abstract] OR "ischemic stroke*"[Title/Abstract] OR "stroke, ischemic"[Title/Abstract] OR "ischaemic stroke*"[Title/Abstract] OR "stroke, ischaemic"[Title/Abstract] OR "acute ischemic stroke"[Title/Abstract] OR "acute IS"[Title/Abstract] OR "acute ISs"[Title/Abstract] OR "cryptogenic ischemic stroke*"[Title/Abstract] OR "cryptogenic stroke*"[Title/Abstract] OR "cryptogenic embolism stroke*"[Title/Abstract] OR "wake up stroke*"[Title/Abstract] OR "brain ischemia*"[Title/Abstract] OR "cerebral ischemia*"[Title/Abstract] OR "brain ischaemia"[Title/Abstract] OR "ischemic encephalopathy"[Title/Abstract] OR "cerebral infarction*"[Title/Abstract] OR "cerebral infarct*"[Title/Abstract] OR "brain infarction*"[Title/Abstract] OR "brain infarct*"[Title/Abstract] OR "intracranial embolism"[Title/Abstract] OR "cerebral embolism"[Title/Abstract] OR "cerebral emboli"[Title/Abstract] OR "cerebral embolus"[Title/Abstract] OR "brain embolism"[Title/Abstract] OR "brain emboli"[Title/Abstract] OR "brain embolus"[Title/Abstract] OR "cerebral stem ischemia"[Title/Abstract] OR "cerebral ischemia reperfusion"[Title/Abstract] OR "reperfusion injury"[Title/Abstract] OR "reperfusion damage"[Title/Abstract])) AND ("cannabinoid*"[Title/Abstract] OR "cannabidiol"[Title/Abstract] OR "epidiolex"[Title/Abstract] OR "CBD"[Title/Abstract] OR "epidyolex"[Title/Abstract] OR "dronabinol"[Title/Abstract] OR "tetrahydrocannabinol"[Title/Abstract] OR "THC"[Title/Abstract] OR "marinol"[Title/Abstract] OR "delta(9) THC"[Title/Abstract] OR "delta(1) tetrahydrocannabinol"[Title/Abstract] OR "delta(1) THC"[Title/Abstract] OR "delta(9) tetrahydrocannabinol"[Title/Abstract] OR "syndros"[Title/Abstract] OR "cannabinol"[Title/Abstract] OR "nabilone"[Title/Abstract] OR "cesamet"[Title/Abstract] OR "canemes"[Title/Abstract] OR "HU 211"[Title/Abstract] OR "dexanabinol"[Title/Abstract] OR "HU 210"[Title/Abstract] OR "nabiximols"[Title/Abstract] OR "sativex"[Title/Abstract] OR "medical marijuana"[Title/Abstract] OR "medicinal marijuana"[Title/Abstract] OR "medical cannabis"[Title/Abstract] OR "medicinal cannabis"[Title/Abstract] OR "medical cannabinoid*"[Title/Abstract] OR "cannabigerol"[Title/Abstract] OR "cannabichromene"[Title/Abstract] OR "cannabidivarin"[Title/Abstract] OR "exocannabinoid"[Title/Abstract] OR "phytocannabinoid*"[Title/Abstract] OR "phyto-cannabinoid*"[Title/Abstract])) | 564 |
| Embase | #1 ('cerebrovascular accident'/exp OR 'cerebrovascular accident') AND [embase]/lim AND [2000-2025]/py OR (('ischemic stroke'/exp OR 'ischemic stroke') AND [embase]/lim AND [2000-2025]/py) OR (('brain ischemia'/exp OR 'brain ischemia') AND [embase]/lim AND [2000-2025]/py) OR (('brain infarction'/exp OR 'brain infarction') AND [embase]/lim AND [2000-2025]/py) OR (('brain embolism'/exp OR 'brain embolism') AND [embase]/lim AND [2000-2025]/py)  #2 (stroke*:ab,ti OR 'cerebrovascular accident*':ab,ti OR 'cerebral stroke*':ab,ti OR 'cerebrovascular apoplexy':ab,ti OR apoplexy:ab,ti OR 'brain vascular accident':ab,ti OR 'ischemic stroke*':ab,ti OR 'stroke, ischemic':ab,ti OR 'ischaemic stroke*':ab,ti OR 'stroke, ischaemic':ab,ti OR 'acute ischemic stroke':ab,ti OR 'acute is':ab,ti OR 'acute iss':ab,ti OR 'cryptogenic ischemic stroke*':ab,ti OR 'cryptogenic stroke*':ab,ti OR 'cryptogenic embolism stroke*':ab,ti OR 'wake up stroke*':ab,ti OR 'brain ischemia*':ab,ti OR 'cerebral ischemia*':ab,ti OR 'brain ischaemia':ab,ti OR 'ischemic encephalopathy':ab,ti OR 'cerebral infarction*':ab,ti OR 'cerebral infarct*':ab,ti OR 'brain infarction*':ab,ti OR 'brain infarct*':ab,ti OR 'intracranial embolism':ab,ti OR 'cerebral embolism':ab,ti OR 'cerebral emboli':ab,ti OR 'cerebral embolus':ab,ti OR 'brain embolism':ab,ti OR 'brain emboli':ab,ti OR 'brain embolus':ab,ti OR 'cerebral stem ischemia':ab,ti OR 'cerebral ischemia reperfusion':ab,ti OR 'reperfusion injury':ab,ti OR 'reperfusion damage':ab,ti) AND [embase]/lim AND [2000-2025]/py  #3 #1 OR #2  #4 ('cannabinoid'/exp OR 'cannabinoid') AND [embase]/lim AND [2000-2025]/py OR (('cannabidiol'/exp OR 'cannabidiol') AND [embase]/lim AND [2000-2025]/py) OR (('dronabinol'/exp OR 'dronabinol') AND [embase]/lim AND [2000-2025]/py) OR (('cannabinol'/exp OR 'cannabinol') AND [embase]/lim AND [2000-2025]/py) OR (('nabilone'/exp OR 'nabilone') AND [embase]/lim AND [2000-2025]/py) OR (('dexanabinol'/exp OR 'dexanabinol') AND [embase]/lim AND [2000-2025]/py) OR (('nabiximols'/exp OR 'nabiximols') AND [embase]/lim AND [2000-2025]/py) OR (('medical cannabis'/exp OR 'medical cannabis') AND [embase]/lim AND [2000-2025]/py) OR (('tetrahydrocannabinol'/exp OR 'tetrahydrocannabinol') AND [embase]/lim AND [2000-2025]/py) OR (('cannabigerol'/exp OR 'cannabigerol') AND [embase]/lim AND [2000-2025]/py) OR (('cannabichromene'/exp OR 'cannabichromene') AND [embase]/lim AND [2000-2025]/py) OR (('cannabidivarin'/exp OR 'cannabidivarin') AND [embase]/lim AND [2000-2025]/py)  #5 (cannabinoid*:ab,ti OR cannabidiol:ab,ti OR epidiolex:ab,ti OR cbd:ab,ti OR epidyolex:ab,ti OR dronabinol:ab,ti OR tetrahydrocannabinol:ab,ti OR thc:ab,ti OR marinol:ab,ti OR 'delta9 thc':ab,ti OR 'delta1 tetrahydrocannabinol':ab,ti OR 'delta1 thc':ab,ti OR 'delta9 tetrahydrocannabinol':ab,ti OR syndros:ab,ti OR cannabinol:ab,ti OR nabilone:ab,ti OR cesamet:ab,ti OR canemes:ab,ti OR 'hu 211':ab,ti OR dexanabinol:ab,ti OR 'hu 210':ab,ti OR nabiximols:ab,ti OR sativex:ab,ti OR 'medical marijuana':ab,ti OR 'medicinal marijuana':ab,ti OR 'medical cannabis':ab,ti OR 'medicinal cannabis':ab,ti OR 'medical cannabinoid*':ab,ti OR cannabigerol:ab,ti OR cannabichromene:ab,ti OR cannabidivarin:ab,ti OR exocannabinoid:ab,ti OR phytocannabinoid*:ab,ti OR 'phyto cannabinoid*':ab,ti)  #6 #4 OR #5  #7 #3 AND #6 | 2409 |
| Cochrane Library | #1 MeSH descriptor: [Stroke] explode all trees  #2 MeSH descriptor: [Ischemic Stroke] explode all trees  #3 MeSH descriptor: [Brain Ischemia] explode all trees  #4 MeSH descriptor: [Cerebral Infarction] explode all trees  #5 MeSH descriptor: [Brain Infarction] explode all trees  #6 MeSH descriptor: [Intracranial Embolism] explode all trees  #7 #1 OR #2 OR #3 OR #4 OR #5 OR #6  #8 ((stroke*) OR (cerebrovascular NEXT accident*) OR (cerebral NEXT stroke*) OR "cerebrovascular apoplexy" OR "apoplexy" OR "brain vascular accident" OR (ischemic NEXT stroke*) OR "stroke, ischemic" OR (ischaemic NEXT stroke*) OR "stroke, ischaemic" OR "acute ischemic stroke" OR "acute IS" OR "acute ISs" OR (cryptogenic NEXT ischemic NEXT stroke*) OR (cryptogenic NEXT stroke*) OR (cryptogenic NEXT embolism NEXT stroke*) OR (wake NEXT up NEXT stroke*) OR (brain NEXT ischemia*) OR (cerebral NEXT ischemia*) OR "brain ischaemia" OR "ischemic encephalopathy" OR (cerebral NEXT infarction*) OR (cerebral NEXT infarct*) OR (brain NEXT infarction*) OR (brain NEXT infarct*) OR "intracranial embolism" OR "cerebral embolism" OR "cerebral emboli" OR "cerebral embolus" OR "brain embolism" OR "brain emboli" OR "brain embolus" OR "cerebral stem ischemia" OR "cerebral ischemia reperfusion" OR "reperfusion injury" OR "reperfusion damage"):ti,ab,kw (Word variations have been searched)  #9 #7 OR #8  #10 MeSH descriptor: [Cannabinoids] explode all trees  #11 MeSH descriptor: [Cannabidiol] explode all trees  #12 MeSH descriptor: [Dronabinol] explode all trees  #13 MeSH descriptor: [Cannabinol] explode all trees  #14 MeSH descriptor: [Medical Marijuana] explode all trees  #15 #10 OR #11 OR #12 OR #13 OR #14  #16 ((cannabinoid*) OR "cannabidiol" OR "epidiolex" OR "CBD" OR "epidyolex" OR "dronabinol" OR "tetrahydrocannabinol" OR "THC" OR "marinol" OR "delta(9) THC" OR "delta(1) tetrahydrocannabinol" OR "delta(1) THC" OR "delta(9) tetrahydrocannabinol" OR "syndros" OR "cannabinol" OR "nabilone" OR "cesamet" OR "canemes" OR "HU 211" OR "dexanabinol" OR "HU 210" OR "nabiximols" OR "sativex" OR "medical marijuana" OR "medicinal marijuana" OR "medical cannabis" OR "medicinal cannabis" OR (medical NEXT cannabinoid*) OR "cannabigerol" OR "cannabichromene" OR "cannabidivarin" OR "exocannabinoid" OR (phytocannabinoid*) OR (phyto-cannabinoid*)):ti,ab,kw (Word variations have been searched)  #17 #15 OR #16  #18 #9 AND #17 | 32 |
| Scopus | TITLE-ABS-KEY (("stroke" OR "apoplexy" OR "ischemic stroke*" OR "ischaemic stroke*" OR "brain ischemia" OR "cerebral ischemia" OR "cerebral infarct" OR "brain infarct" OR "intracranial embolism" OR "cerebrovascular accident" OR "brain vascular accident" OR "cerebral infarction" OR "brain ischaemia" OR "brain infarction" OR "ischemic encephalopathy" OR "stroke, ischemic" OR "stroke, ischaemic" OR "acute IS" OR "acute ISs" OR "cerebral stem ischemia" OR "acute ischemic stroke" OR "cryptogenic ischemic stroke" OR "cryptogenic stroke" OR "cryptogenic embolism stroke" OR "wake up stroke" OR "cerebral ischemia reperfusion" OR "reperfusion injury" OR "reperfusion damage" OR "cerebral embolism" OR "cerebral emboli" OR "cerebral embolus" OR "brain embolism" OR "brain emboli" OR "brain embolus") AND ("cannabinoid*" OR "cannabidiol" OR "tetrahydrocannabinol" OR "dronabinol" OR "CBD" OR "THC" OR "epidiolex" OR "epidyolex" OR "marinol" OR "syndros" OR "cannabinol" OR "nabilone" OR "cesamet" OR "canemes" OR "dexanabinol" OR "nabiximols" OR "sativex" OR "cannabigerol" OR "cannabichromene" OR "HU 210" OR "HU 211" OR "cannabidivarin" OR "medical marijuana" OR "medical cannabis" OR "medicinal marijuana" OR "medicinal cannabis" OR "medical cannabinoid*" OR "exocannabinoid*" OR "phytocannabinoid*" OR "phyto-cannabinoid*" OR "delta(9) THC" OR "delta(1) tetrahydrocannabinol" OR "delta(1) THC" OR "delta(9) tetrahydrocannabinol")) | 1297 |

**Supplementary Table 2:** Risk of bias for inclusion of studies.

| **Study** | **A** | **B** | **C** | **D** | **E** | **F** | **G** | **H** | **I** | **J** |
| --- | --- | --- | --- | --- | --- | --- | --- | --- | --- | --- |
| Villa (2024a) | + | + | ※ | ※ | ※ | ※ | + | + | + | + |
| Chen (2024) | ※ | + | ※ | ※ | + | ※ | + | + | + | + |
| Xu (2023) | + | + | ※ | ※ | + | ※ | + | + | + | + |
| Lavayen (2023) | ※ | + | ※ | ※ | ※ | ※ | ※ | + | + | + |
| Meyer (2022) | + | + | ※ | ※ | ※ | ※ | ※ | + | + | + |
| Khaksar (2022) | + | + | ※ | ※ | ※ | ※ | ※ | + | + | + |
| Yokubaitis (2021) | ※ | + | ※ | ※ | ※ | ※ | ※ | + | + | + |
| Rodríguez-Muñoz (2018) | + | + | ※ | ※ | + | ※ | ※ | + | + | + |
| Khaksar (2017a) | + | + | ※ | ※ | ※ | ※ | ※ | + | + | ※ |
| Khaksar (2017b) | + | + | ※ | ※ | ※ | ※ | ※ | + | + | ※ |
| Ceprian (2017) | + | + | ※ | ※ | ※ | ※ | + | + | + | + |
| Villa (2024b) | + | + | ※ | ※ | ※ | ※ | + | + | + | + |
| Liu (2022) | ※ | + | ※ | ※ | ※ | ※ | ※ | + | + | + |
| Khaksar (2017c) | + | + | ※ | ※ | ※ | ※ | ※ | + | + | ※ |
| Hayakawa (2009) | ※ | + | ※ | ※ | ※ | ※ | ※ | + | + | + |
| Hayakawa (2008) | ※ | + | ※ | ※ | ※ | ※ | ※ | + | + | + |
| Durmaz (2008) | ※ | + | ※ | ※ | ※ | ※ | ※ | + | + | + |
| Hayakawa (2007a) | ※ | + | ※ | ※ | ※ | ※ | ※ | + | + | + |
| Hayakawa (2007b) | ※ | + | ※ | ※ | ※ | ※ | ※ | + | + | + |
| Hayakawa (2007c) | ※ | + | ※ | ※ | ※ | ※ | ※ | + | + | + |
| Mishima (2005) | ※ | + | ※ | ※ | ※ | ※ | ※ | + | + | + |
| Hayakawa (2004) | ※ | + | ※ | ※ | ※ | ※ | ※ | + | + | + |
| Teichner (2003) | ※ | + | ※ | ※ | ※ | ※ | ※ | + | + | + |
| Leker (2003) | ※ | + | ※ | ※ | ※ | ※ | + | + | + | + |
| Lavie (2001) | ※ | + | ※ | ※ | ※ | ※ | + | + | + | + |
| de Souza Stork (2025) | + | + | ※ | ※ | ※ | ※ | + | + | + | + |

A, Random sequence generation (selection bias); B, Baseline characteristics (selection bias); C, Allocation concealment (selection bias); D, Random housing (performance bias); E, Blinding of participants and personnel (performance bias); F, Random of outcome assessment (detection bias); G, Blinding of outcome assessment (detection bias); H, Incomplete outcome data (attrition bias); I, Selective reporting (reporting bias); J, Other bias. +: low risk; ※: unclear risk.

**Supplementary Table 3:** Subgroup analysis of CBF.

| **Subgroup** | **Study** | **SMD (95% CI)** | **Heterogeneity *I^2^* (%)** | ***P* value** | **Subgroup (*P* value)** |
| --- | --- | --- | --- | --- | --- |
| Drug class |  |  |  |  | 0.002 |
| CBD | 3 | 10.29 (4.42, 16.15) | 0 | 0.89 |  |
| HU-210 | 1 | 0.00 (-1.24, 1.24) | - | - |  |
| THC | 2 | 1.65 (-0.02, 3.32) | 0 | 0.41 |  |
| Route of administration |  |  |  |  | 0.02 |
| Ip | 5 | 4.13 (0.77, 7.50) | 54 | 0.07 |  |
| Iv | 1 | 0.00 (-1.24, 1.24) | - | - |  |
| Timing of administration |  |  |  |  | 0.07 |
| Pre-MCAO | 2 | 5.29 (-5.54, 16.11) | 69 | 0.07 |  |
| Post-MCAO | 1 | 0.00 (-1.24, 1.24) | - | - |  |
| Pre- and post-MCAO | 3 | 5.62 (0.54, 10.70) | 40 | 0.19 |  |

CBD, cannabidiol; THC, D-9-tetrahydrocannabinol; ip, intraperitoneal injection; iv, intravenous injection; MCAO, middle cerebral artery occlusion.

**Supplementary Table 4:** Subgroup analysis of TUNEL-positive cells.

| **Subgroup** | **Study** | **SMD (95% CI)** | **Heterogeneity *I^2^* (%)** | ***P* value** | **Subgroup (*P* value)** |
| --- | --- | --- | --- | --- | --- |
| Drug class |  |  |  |  | 0.51 |
| CBD | 2 | -1.90 (-3.96, 0.17) | 82 | 0.02 |  |
| HU-211 | 1 | -2.67 (-4.62, -0.72) | - | - |  |
| VCE-004.8 | 1 | -1.37 (-2.45, -0.28) | - | - |  |
| Route of administration |  |  |  |  | 0.35 |
| Ip | 3 | -1.62 (-2.69, -0.55) | 64 | 0.06 |  |
| Iv | 1 | -2.67 (-4.62, -0.72) | - | - |  |
| Timing of administration |  |  |  |  | 0.35 |
| Pre-MCAO | 1 | -2.67 (-4.62, -0.72) | - | - |  |
| Post-MCAO | 3 | -1.62 (-2.69, -0.55) | 64 | 0.06 |  |

CBD, cannabidiol; ip, intraperitoneal injection; iv, intravenous injection; MCAO, middle cerebral artery occlusion.

**Supplementary Table 5:** Subgroup analysis of TNF-α level.

| **Subgroup** | **Study** | **SMD (95% CI)** | **Heterogeneity *I^2^* (%)** | ***P* value** | **Subgroup (*P* value)** |
| --- | --- | --- | --- | --- | --- |
| Animal species |  |  |  |  | 0.46 |
| SD rats | 3 | -1.19 (-2.12, -0.26) | 0 | 0.51 |  |
| Wistar rats | 3 | -2.23 (-4.78, 0.33) | 84 | 0.002 |  |
| Anesthetic agent |  |  |  |  | 0.09 |
| Isoflurane | 1 | -2.51 (-5.42, 0.39) | - | - |  |
| Phenobarbital | 2 | -1.04 (-2.02, -0.06) | 0 | 0.49 |  |
| Sevoflurane | 1 | -0.19 (-1.06, 0.68) | - | - |  |
| Other | 2 | -3.56 (-6.64, -0.48) | 64 | 0.10 |  |
| Drug class |  |  |  |  | 0.02 |
| CBD | 5 | -1.97 (-3.18, -0.75) | 49 | 0.10 |  |
| VCE-004.8 | 1 | -0.19 (-1.06, 0.68) | - | - |  |
| Route of administration |  |  |  |  | 0.30 |
| Icv | 2 | -3.56 (-6.64, -0.48) | 64 | 0.10 |  |
| Ip | 2 | -0.92 (-3.03, 1.20) | 56 | 0.13 |  |
| Iv | 2 | -1.04 (-2.02, -0.06) | 0 | 0.49 |  |
| Timing of administration |  |  |  |  | 0.08 |
| Pre-MCAO | 2 | -3.56 (-6.64, -0.48) | 64 | 0.10 |  |
| Post-MCAO | 4 | -0.75 (-1.50, 0.01) | 20 | 0.29 |  |

SD rats, Sprague-Dawley rats; CBD, cannabidiol; ip, intraperitoneal injection; icv, intraventricular injection; iv, intravenous injection; MCAO, middle cerebral artery occlusion.


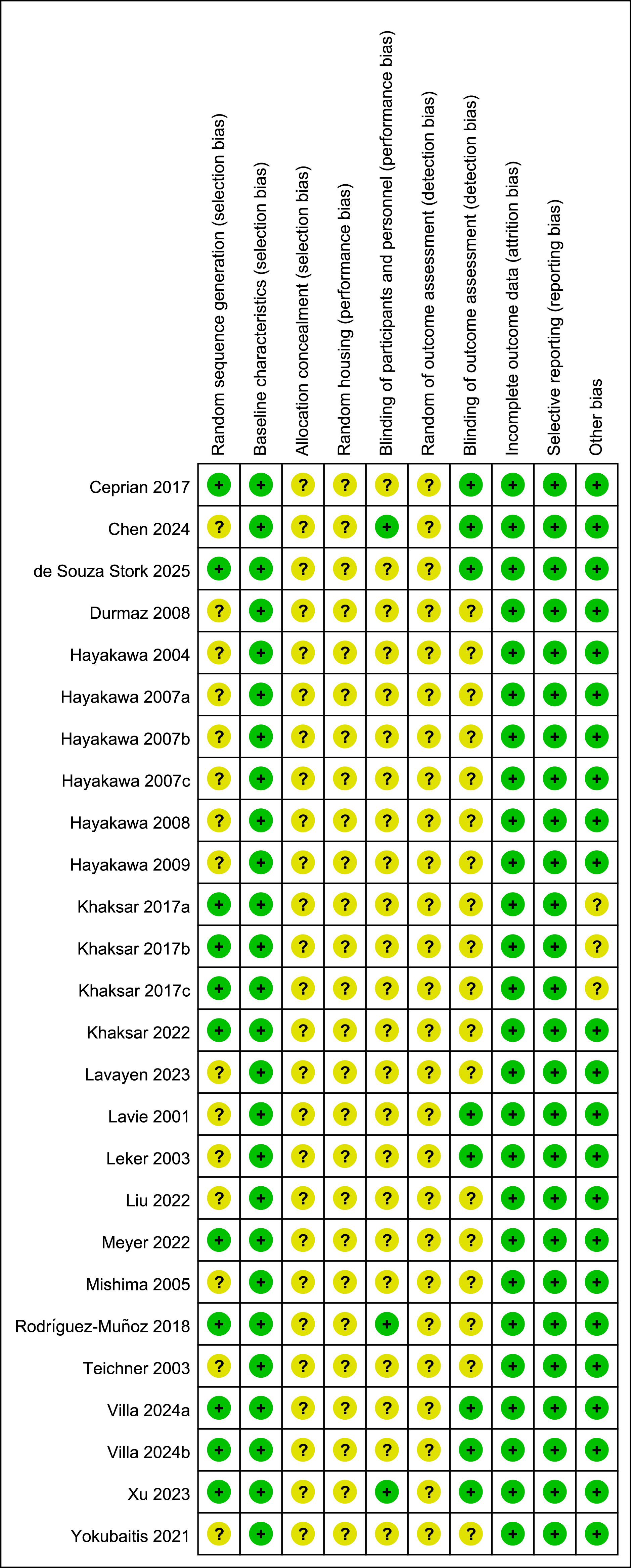


**Supplementary Figure 1.** Summary of risk of bias assessment for included studies. +, low risk; ?, unclear risk.


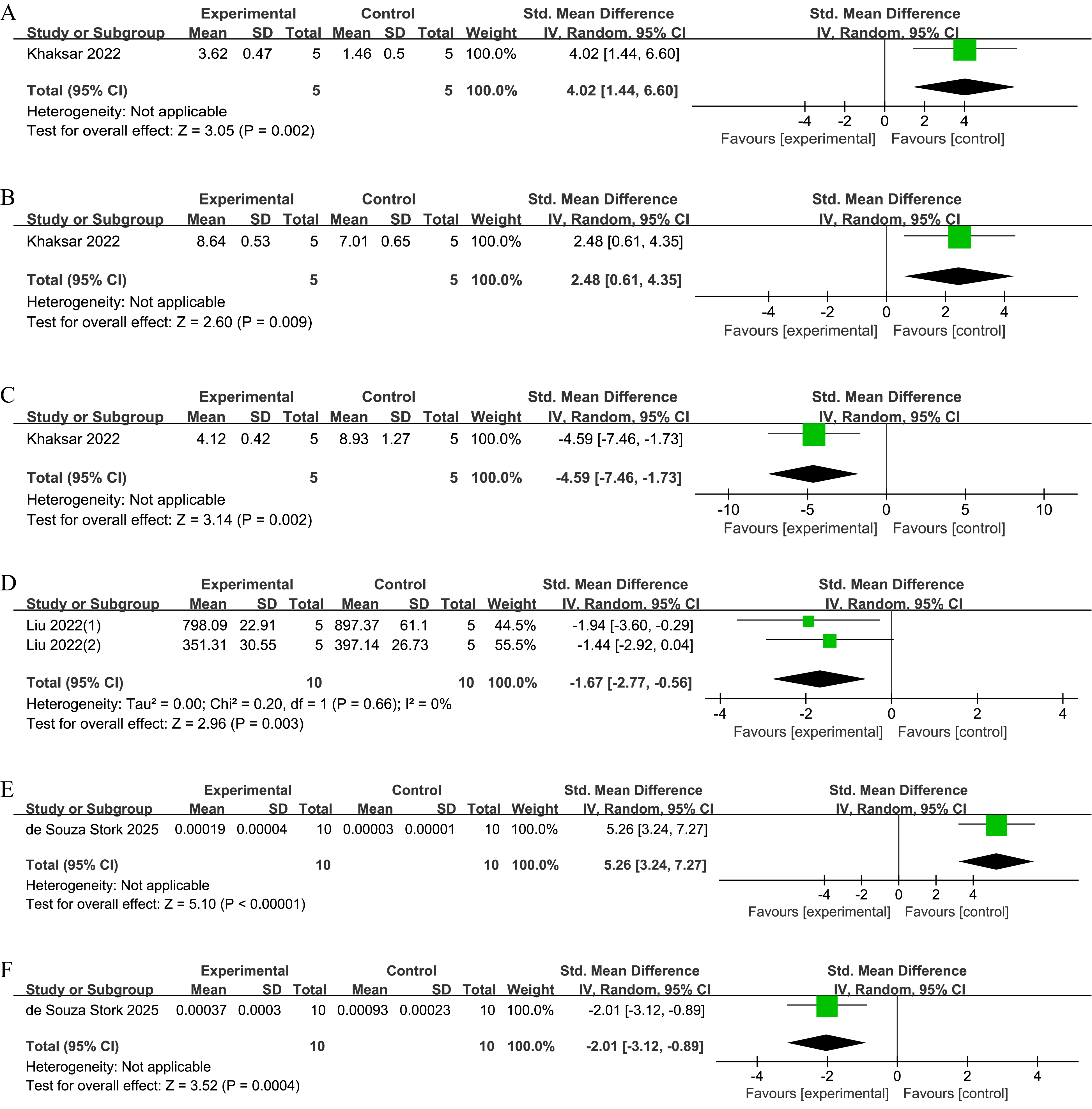


**Supplementary Figure 2.** Forest plot illustrating the effects of MCs on oxidative stress markers. (A) Forest plot of SOD. (B) Forest plot of CAT. (C) Forest plot of MDA. (D) Forest plot of ROS. (E) Forest plot of CAT in lung tissue. (F) Forest plot of MDA in lung tissue.


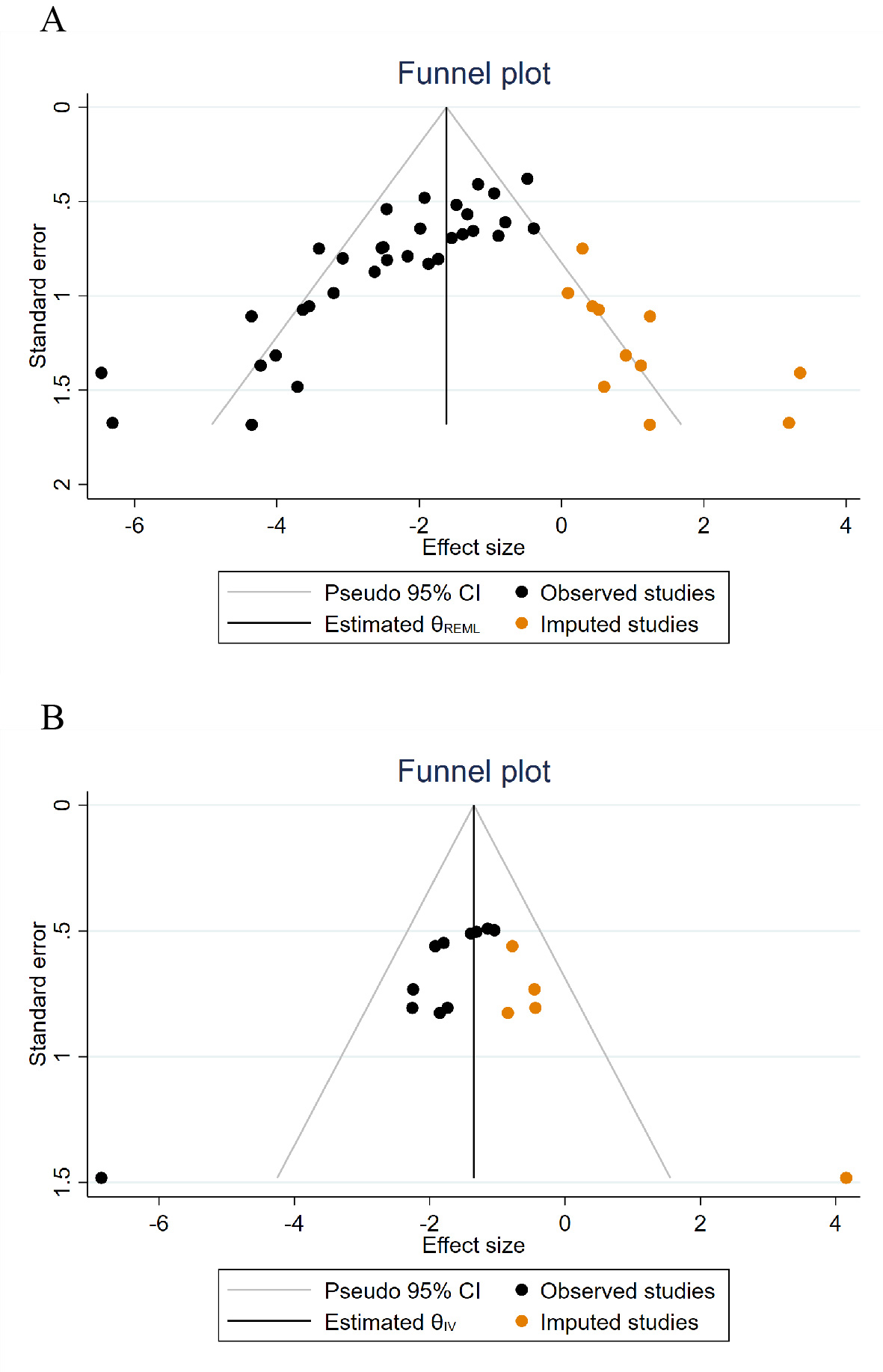


**Supplementary Figure 3.** (A) Trim-and-fill analysis of cerebral infarct volume. (B) Trim-and-fill analysis of neurological function score.


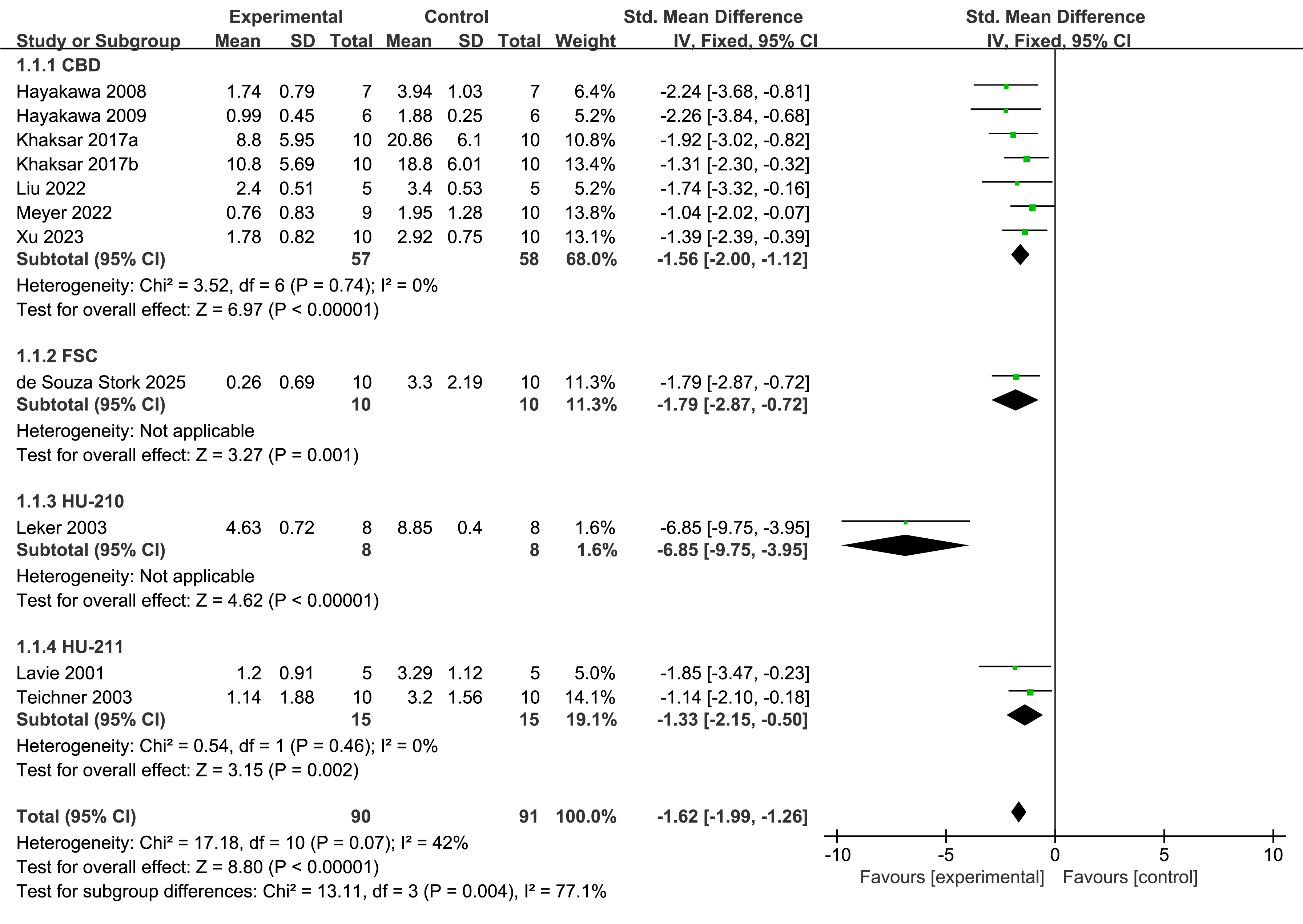


**Supplementary Figure 4.** Forest plot illustrating the effects of MCs on neurological function score.
